# A Systematic Review and Meta-Analysis of the Potential of Millets for Managing and Reducing the Risk of Developing Diabetes Mellitus

**DOI:** 10.3389/fnut.2021.687428

**Published:** 2021-07-28

**Authors:** Seetha Anitha, Joanna Kane-Potaka, Takuji W. Tsusaka, Rosemary Botha, Ananthan Rajendran, D. Ian Givens, Devraj J. Parasannanavar, Kowsalya Subramaniam, Kanaka Durga Veera Prasad, Mani Vetriventhan, Raj Kumar Bhandari

**Affiliations:** ^1^Smart Food Initiative, International Crops Research Institute for the Semi-Arid Tropics (ICRISAT), Patancheru, India; ^2^Organization for Advanced and Integrated Research, Kobe University, Kobe, Japan; ^3^Development Strategy and Governance Division, International Food Policy Research Institute (IFPRI), Lilongwe, Malawi; ^4^National Institute of Nutrition (NIN), Hyderabad, India; ^5^Institute of Food, Nutrition and Health, University of Reading, Reading, United Kingdom; ^6^Food Science and Nutrition, Avinashilingam Institute for Home Science and Higher Education for Women, Coimbatore, India; ^7^National Technical Board of Nutrition, Government of India (GoI), New Delhi, India

**Keywords:** millets, sorghum, diabetes, glycaemic index, glycaemic response, meta-analaysis

## Abstract

Millets (including sorghum) are known to be highly nutritious besides having a low carbon footprint and the ability to survive in high temperatures with minimal water. Millets are widely recognised as having a low Glycaemic Index (GI) helping to manage diabetes. This systematic review and meta-analyzes across the different types of millets and different forms of processing/cooking collated all evidences. Of the 65 studies that were collected globally, 39 studies with 111 observations were used to analyze GI outcomes and 56 studies were used to analyze fasting, post-prandial glucose level, insulin index and HbA1c outcomes in a meta-analysis. It is evident from the descriptive statistics that the mean GI of millets is 52.7 ± 10.3, which is about 36% lower than in typical staples of milled rice (71.7 ± 14.4) and refined wheat (74.2 ± 14.9). The descriptive, meta and regression analyses revealed that Job's tears, fonio, foxtail, barnyard, and teff were the millets with low mean GI (<55) that are more effective (35–79%) in reducing dietary GI than the control samples. Millets with intermediate GI (55–69) are pearl millet, finger millet, kodo millet, little millet, and sorghum which have a 13–35% lower GI than the control with high GI (>69). A meta-analysis also showed that all millets had significantly (*p* < 0.01) lower GI than white rice, refined wheat, standard glucose or white wheat bread except little millet which had inconsistent data. Long term millet consumption lowered fasting and post-prandial blood glucose levels significantly (*p* < 0.01) by 12 and 15%, respectively, in diabetic subjects. There was a significant reduction in HbA1c level (from 6.65 ± 0.4 to 5.67 ± 0.4%) among pre-diabetic individuals (*p* < 0.01) who consumed millets for a long period. Minimally processed millets were 30% more effective in lowering GI of a meal compared to milled rice and refined wheat. In conclusion, millets can be beneficial in managing and reducing the risk of developing diabetes and could therefore be used to design appropriate meals for diabetic and pre-diabetic subjects as well as for non-diabetic people for a preventive approach.

## Introduction

It is estimated that there will be a 51% surge in diabetics globally by 2045, from 463 million in 2019 to 700 million in 2045 (1) with type 2 diabetes accounting for about 90% of the total. Eighty-seven percent of diabetes-related deaths occur in low and middle income countries where there is less diversification of staple foods. It is important to note that apart from a sedentary lifestyle and obesity, the type of food consumed plays a key role in diabetes. Main staples such as refined rice, refined wheat and maize contribute up to 80% of the energy intake in developing countries (2). Diversifying food staples and mainstreaming traditional nutritious and less glucogenic staples in the majority of developing countries is very important to manage and prevent diabetes; millets and sorghum figure first in this list of staples.

The value of a Triple Bottom Line is well-recognised in businesses and has been the stimulus for the creation of new products and impactful investments. Customising it to the Food System is the Smart Food Triple Bottom Line, defining solutions (3) that in unison are good for you (nutritious and healthy), good for the planet (environmentally sustainable) and good for the farmer (resilient). It is an approach being used to analyze the value of millets and sorghum as staples. This is the first analyses focusing on how millets and sorghum are “good for you” in terms of reducing diabetes, and comparing them to rice, wheat and maize, the “Big 3” major staple foods in Asia and Africa. Of these, polished rice, which is inherently deficient in micronutrients, provides 80% of the energy intake (4) in high rice consuming countries. Growing lifestyle diseases like type 2 diabetes make it imperative to explore dietary solutions that include nutrition and tackle major health issues. Diversifying diets by diversifying staples with the right nutritious and healthy foods can play a major role in reducing multiple health related burdens.

There are 13 types of millets available globally (5) which include pearl millet, finger millet, sorghum, little millet, proso millet, kodo millet, barnyard millet, brown top millet, foxtail millet, Guinea millet, Job's tears, fonio, and teff. Except for Job's tears, fonio, and teff, the other millets are widely distributed in India. Finger millet is widely found in India, China and in some Eastern and Southern African countries, whereas fonio is widely distributed in Western Africa and Job's tears in northeast India, southern and eastern Asia and southern China. On the other hand, teff is mainly found in Ethiopia (5). Currently, these crops are mostly grown in Africa and Asia as well as in the USA, which is the largest producer of sorghum. Millets also occur in other parts of the world as feed and fodder or as a minor crop (www.smartfood.org/millets-sorghum-production-trends/).

A systematic review of 19 research articles showed that millets help manage diabetes due to their high fibre, polyphenol, and antioxidant content (6). Millets were traditionally consumed in African and Asian countries and were later largely replaced by rice, wheat and maize. Considering nutrient requirements, rising non-communicable health issues like diabetes and challenges posed by climate change, it is important to popularise smart foods, i.e., foods that fulfil all criteria of being good for you, the planet and the farmer.

Many studies have demonstrated the efficacy of millets in improving glycaemic control, decreasing fasting, and post-prandial rise in blood glucose concentration (7, 8), reducing insulin index and insulin resistance and lessening glycosylated haemoglobin (HbA1c) level (8–12). Glycaemic index (GI) is a measure of how much the carbohydrate present in the food affects the rate and extent of change in post-prandial blood glucose concentration. The general dietary strategy to enhance glycaemic control is to consume low GI food (13). Fasting blood glucose is generally measured following overnight fasting and post-prandial blood glucose is measured at regular intervals of up to 2 h after eating. Hyperinsulinemia is associated with insulin resistance that increases the risk of type 2 diabetes (14). Therefore, along with post-prandial glucose concentration, it is important to measure insulin concentration in order to evaluate a food's ability to reduce insulin resistance. In addition, long term glycaemic control can be measured by HbA1c marker (15).

Although there are several studies on millets related to these outcomes, their information is heterogeneous. Therefore, it is important to collate scientific evidence to determine whether the studies support the glycaemic controlling ability of millets or not, including all the types and forms of processing (including cooking) they undergo, in order to serve as a dietary guide on millets. Considering the growing prevalence of diabetes among high and low socioeconomic groups in both developed and developing countries, this paper for the first time aims to undertake an in-depth systematic review and meta-analysis, simple descriptive statistics, and regression analysis of all the studies conducted to test GI, fasting and post-prandial blood glucose concentrations, insulin response and HbA1c biomarker level in millet-based diets. This includes 11 types of millets, 1 mixed millet and many forms of processing that were tested. This information will form the scientific basis for any claims about millets vis-à-vis diabetes and be useful for the scientific community, dieticians, and nutritionists through to food processors and governments in setting policies and programs on health, nutrition and agriculture. Therefore, this study aims to address the following research question:

Does consuming millet(s)-based food help in managing and reducing the risk of developing type 2 diabetes compared to the consumption of typical staples?

## Methods

The systematic review was conducted by: (1) collating all the relevant studies on the glucogenic effect of millets relative to other staple foods; (2) reviewing the methods used to study this; (3) conducting a regression analysis to find the effect of millets in managing diabetes and (4) conducting a meta-analysis to assess the science-based evidence on millets' ability to reduce insulin concentration, HbA1c biomarker and fasting and post-prandial blood glucose concentration and their effect on managing individuals with type 2 diabetes mellitus and pre-diabetic individuals compared to non-millet-based regular diets or other staples.

The following sections describe the methods in detail.

### Study Period and Protocol

The systematic review was conducted from October 2017 to February 2021. The study protocol is registered in the Research Registry (Unique Identification Number; reviewregistry1094) and a 27-item PRISMA checklist was used to conduct the systematic review and meta-analysis (16).

### Search, Inclusion, and Exclusion Criteria

The search basically selected all the research studies in English conducted from the year 1950 to the last quarter of 2020. An initial scoping study was conducted using PubMed and MEDLINE to check for studies that overlapped with the research question of the systematic review as per the guidelines of Atkinson and Cipriani, 2018 (17). Later, a detailed search was conducted using search engines Google scholar, Scopus, Web of Science, PubMed (MEDLINE), CAB Abstracts ClinicalTrials.gov, grey literature, and other Clinical Trial Registries to find the studies relevant to the research question. The search was conducted using the search strategy and keywords indicated in Table 1, with further screening for study relevance, completeness of information and quality of research based on the inclusion and exclusion criteria.

**Table 1:** Search strategy and keywords used to identify the relevant papers.

**Number**	**Criteria and keywords used for the search**
1	Boolean logic such as “AND,” “OR,” “NOT” were used
2	Finger millet glycaemic index. Repeat the search by replacing finger millet with other millets in the following list: “little millet,” “foxtail millet,” “barnyard millet,” “proso millet,” “kodo millet,” “teff,” “fonio,” “job's tears,” “pearl millet,” “finger millet,” and “sorghum”
3	Common name or local name of the millets. For example: adlay (job's tears), acha (fonio), samai (little millet), and navane (foxtail millet)
4	Glucose response of millets. Glycaemic Load (GL) of millets
5	Glucose response of finger millet. Repeated the search with all the millets in the list
6	Glucose lowering effect of finger millet. Repeated the search with all the millets in the list
7	Effect of finger millet on diabetes. Repeated the search with all the millets in the list
8	Effect of finger millet in managing diabetes. Repeated the search with all the millets in the list
9	Effect of millets on fasting blood glucose level. Repeated the search with all the millets in the list
10	Effect of millets on post-prandial blood glucose level. Repeated the search with all the millets in the list
11	Effect of millets on the insulin index. Repeated the search with all the millets in the list
12	Effect of millets on HbA1c or glycosylated haemoglobin
13	Search by using all the keywords mentioned above along with country and continent
14	A hand search was done using the reference list of one paper to find other papers

### Inclusion Criteria

1. Research studies conducted on humans with all types of millets including sorghum, finger millet, pearl millet, little millet, kodo millet, barnyard millet, foxtail millet, proso millet, teff, fonio, and Job's tears. 2. Where there were no or very few human studies on some millets (only for teff and fonio), *in-vitro* studies were included but these were considered separately. 3. Studies with information on any one or all of the outcomes including GI, fasting, post-prandial glucose level, insulin index and HbA1c of any millets were selected for the next level of screening. 4. A study conducted in any geographical location globally was selected. 5. Both randomised cross-over studies and self-controlled case studies were included. 6. Studies conducted on both normal healthy subjects, pre-diabetic, and type 2 diabetic subjects were included. 7. Only peer-reviewed research articles were selected.

### Exclusion Criteria

These included review articles, animal studies and papers where the full information could not be accessed or if the methodologies were identified as weak. Papers representing glucose response values in figures without providing numeric values were excluded from the meta-analysis.

### Data Collection Process

**Figure 1:**
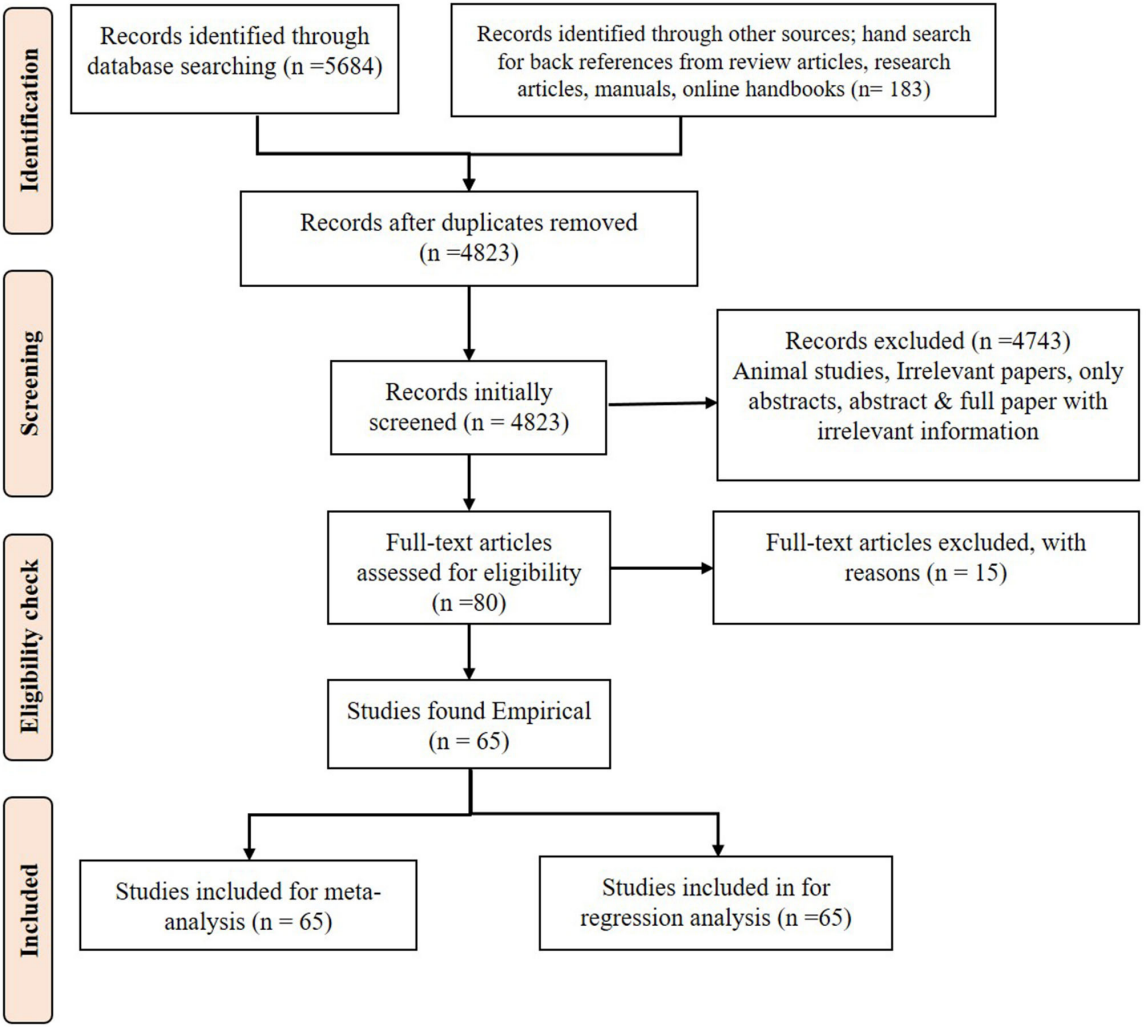
A PRISMA flow diagram of the systematic review.

The PRISMA flow diagram (Figure 1) shows the study design and the criteria for including and excluding papers. Only relevant papers that addressed the research questions were downloaded. If only the abstract was suitable, then open access articles were downloaded, and the full paper was collected by contacting the authors, editors of the journals, universities that have library facilities and subscription to the journal. Some full papers were purchased. After collecting the full paper, if any information on GI and/or glucose response was missing, the authors were contacted and complete information was requested for use in the meta-analysis. A manual search was done in every article to find more related research articles. References in the selected articles were also searched and the full articles were acquired and included in the study, where appropriate.

The data were extracted from the articles and documented in an Excel sheet and then used to conduct the regression analysis, forest plots and publication bias plots.

### Study Quality Assessment

Information such as the author's name, year of publication, geographical region of study, name of the study, gender of the subjects, age range, mean age, study type, sample size, dietary assessment methods used, outcomes, level of dietary exposure, and procedures and standards followed to estimate GI, etc., were extracted from the research articles. Using the eight-item Newcastle-Ottawa Scale (NOS), the quality (18, 19) of each study was assessed by two investigators, and any disagreements were resolved by discussing it with a third reviewer. The NOS allows the assessment of a study population and selection with comparable outcomes of interest. The scale ranged from 0 to 9, and studies with scores of <7 were assigned low quality and those >7 were assigned high quality. The researchers also applied the principle of Bell et al. (20) to further strengthen quality assessment.

### Data Items and Extraction

Each study was labelled with details of the author and year. The numerical variables considered for the meta-analysis included mean GI with standard deviation (SD), mean fasting, and post-prandial blood glucose concentration with SD, the sample size in both intervention and control and mean insulin level with SD. The respective control samples for each study were identified and appropriate data were extracted. Control samples included those of wheat, refined wheat, rice (white and brown), roots, tubers, and legumes. When a food control was not used, then data for glucose or white bread were used as the control. The numerical variables corresponding to GI were extracted as mean GI with SD. If mean standard error (SEM or SE) values were provided in the study, then the SE values were converted into SD values. If the GI was not provided in the paper, it was either obtained from the author, or if the mean of all subjects Area Under the Curve (AUC) was available, then GI was calculated using the formula F/R × 100, where F is the mean of all subjects' AUC for the test food and R is the mean of all subjects' AUC for the control food (21). Fasting and post-prandial glucose concentrations were extracted into the Excel sheet in mg/dl units as per the guidelines provided by Harrer et al. (22). Where given as mmol/l, the values were converted into mg/dl to maintain uniformity of data. HbA1c was presented in percentage and taken as such. Categorical data was recorded on cooking method (baking/roasting, boiling, steam cooking), information on the cooked product (pancake, flatbread, porridge, cooked grain), the form of the samples used (grain, flour, batter) and the health condition (diabetic, pre-diabetic, and non-diabetic) of the study participants.

### Summary Measures and Result Synthesis

A meta-analysis was conducted to estimate the standard mean difference (SMD) and associated heterogeneity (*I*^2^) (23). The significance of the result was determined using a fixed effect model for a single source of information and random effect model for other studies. Subgroup analyses were conducted to ascertain the effect of different variables and conditions on fasting and post-prandial glucose levels. In addition, descriptive statistics such as mean, SD for GI, HbA1c, and glucose level were calculated for both intervention and control samples. A regression analysis was conducted to quantify the effect of millets and control samples on glycaemic control keeping GI as a dependent variable and food type, source, processing methods and participants' health (diabetic, pre-diabetic, and non-diabetic) as independent variables.

### Data Analysis in Detail

In total, 80 studies were collected on the effect of millets on various outcomes in non-diabetic, pre-diabetic, and diabetic subjects. Of these, only 65 studies had complete information on either of the five key outcomes (GI, fasting, post-prandial blood glucose concentration, insulin concentration, and HbA1c level). The effects of millets and control samples were analysed by segregating them in several ways, such as by the effect of consuming millets on five key outcomes in diabetic, pre-diabetic, and non-diabetic subjects, and by comparing the effect of millets on five key outcomes with that on various staples segregated as rice (white and brown), wheat (whole and refined), roots and tubers, legumes and others, standard glucose and white bread. Descriptive statistics, regression and meta-analysis were conducted. Descriptive statistics calculated mean, standard deviation and percentage values of outcomes. A meta-analysis was conducted to generate evidence on the effects of millets' use on GI, fasting and post-prandial glucose levels compared to the pre intervention values (baseline) or control samples used in the studies which included rice (refined and brown), refined wheat and maize. Regression analysis was conducted to test the correlation between type of crop, cooking type, and GI. Both descriptive and regression analyses were conducted using STATA 16 (24). A meta-analysis was conducted using software R studio version 3.5.1 (2018) to obtain forest plots and estimates of heterogeneity (*I*^2^) to evaluate the randomisation of the studies.

#### Meta-Analysis

Sixty-five human studies using various types and forms of millets were used for the meta-analysis to create forest plots for GI (112 observations) and glucose levels at 0 min (fasting blood glucose) and 120 min (post-prandial blood glucose) in normal, pre-diabetic, and diabetic subjects. The millets were compared with the corresponding control samples used in the study. The heterogeneity of the samples (*I*^2^), and overall test results were obtained in forest plots along with *p*-values to test the significance of effect. Both the random effect model and fixed effect model were tested and used to interpret the results of each of the five outcomes. Wherever heterogeneity was low (*I*^2^ <50%), a fixed effect model was used to interpret the result. In addition, where there was only a single source of information from the same population, a fixed effect model was used for the interpretation (25).

#### Subgroup Analysis

Three subgroup analyses were undertaken by identifying changes that possibly affect the five outcomes. This was done based on the type of control (glucose, refined wheat based, rice based, whole wheat based, pulses and legumes based, maize/corn based, other cereals based, and others) used in each study, participant's health condition (non-diabetic, pre-diabetic, and type 2 diabetic), and type of millet used in the studies. Note that the age group of the participants was given as the mean age in years in many studies. Hence, a subgroup analysis based on age was not conducted.

### Risk of Bias

Funnel plots were generated to determine publication bias (23, 26). In addition, each study was scored for biases related to selection, performance, detection, attrition and reporting to generate a risk of bias plot.

#### Regression Analysis

Regression analysis is a statistical procedure for estimating the relationships between a dependent variable and independent variables. To quantify the effects of crop choice on GI (in all *in vivo* studies and only 2 *in vitro* studies), ordinary least squares (OLS) regression (27, 28) with cluster-robust standard errors (29) was performed using the metadata including 267 observations from 63 studies. OLS is the most common linear least square method of estimating the coefficient in a linear regression model. Here the dependent variable was the GI value, the main independent variables were a set of dummy variables representing different crops and the control variables were the grain processing and cooking methods, the Type 2 diabetes mellitus condition of the subjects and the method of GI measurement. More specifically, the OLS equation is expressed as follows:

$$\begin{array}{l} y_{i}=\beta_{0}+\overset{17}{\underset{j=1}{\sum }}\beta_{1j}x_{1ji}+\beta_{2}x_{2i}+\overset{3}{\underset{l=1}{\sum }}\beta_{3l}x_{3li}+\beta_{4}x_{4i}+\beta_{5}x_{5i}\\ \;+\overset{63}{\underset{n=1}{\sum }}\beta_{6n}x_{6ni}+\varepsilon_{i} \end{array}$$

where *y*_*i*_ represents the GI value for the observation *i* (*i* = 1, 2, 3, …, 267), β_0_ is the intercept term, *x*_1*ji*_ is the set of 17 dummy variables representing 17 crops compared against maize being the base crop, *x*_2*i*_ is the dummy variable that takes the value of one when the food sample is made from a whole grain and zero otherwise, *x*_3*li*_ is the set of 3 dummies representing 3 cooking methods compared against raw consumption being the base method, *x*_4*i*_ is the dummy that takes one when the subject individual has the type 2 diabetic condition and zero otherwise, *x*_5*i*_ is the dummy that takes one when the GI value was estimated using the *in vitro* digestion rate and zero otherwise, *x*_6*ni*_ (*n* = 1, 2, 3, …, 63) is the set of 63 dummies to control for any literature-specific fixed effects arising from any unobservable factor such as individual-specific food sample preparation practise, researcher-specific GI measurement practise, etc., and ε_*i*_ is the random error term. In addition, the interaction term between the type 2 diabetic condition and crop dummies was also examined.

The 17 crops compared with maize were Job's tears (adlay millet), barnyard millet, finger millet, fonio, foxtail millet, kodo millet, little millet, pearl millet, mixed millet (i.e., a mixture of millets and other crops), sorghum, teff, legume, roots and tubers, rice, refined wheat, wheat-based, and other (any other crops were regarded as one group). The three cooking methods analysed were boiling, steaming, and baking (and/or roasting) which were compared with no cooking. To account for literature-level clustering that results in downward bias in the standard errors stemming from any within-literature correlation, cluster-robust standard errors (29, 30) were adopted to correct for heteroscedasticity.

The most important feature of the multiple regression (there is more than one independent variable) is that the covariates are controlled for in the estimation of the coefficient of a certain variable. In our case, for instance, whether the food was made from whole grain or refined grain was controlled for when estimating the effect of a specific crop on GI. In other words, the estimation process incorporated both whole food and refined food, but only compared it with like variables (whole grain millet vs. whole grain maize, refined millet vs. refined maize, etc.,) where these values are either observed or estimated. Hence, the conclusion only reflects such fair comparisons.

## Results

For the meta-analysis, 65 human studies qualified for the five outcomes (GI, fasting blood glucose, post-prandial blood glucose, insulin level, and HbA1c). Some authors conducted studies on more than one type of millet; therefore, the same author contributed to more than one crop studied. This resulted in the identification of 99 studies from 65 authors, which included 19 studies on finger millet, 20 on foxtail millet, 10 each on sorghum and pearl millet, 7 on barnyard millet, 4 each on little and kodo millet, 3 each on teff, fonio and Job's tears, 1 on proso millet, and 15 on a mix of these millets. Apart from this, there were two *in vitro* studies that were included for teff and fonio, with 11 observations for GI (31, 32).

### Descriptive Statistics

Table 2 shows the mean GI of each millet tested *in vivo* along with refined wheat and milled rice. The overall mean GI of millet, milled rice and refined wheat were 52.7 ± 10.3, 71.7 ± 14.4, and 74.2 ± 14.9, respectively. Except for proso millet, all other millets fell in the low to medium GI food category. Table 2 also shows the *in vitro* GI of two types of millets.

**Table 2:** A comparison of millets' glycaemic index measured *in vivo* with control samples using different statistical analyses.

**Type of millet**	**Mean glycaemic index**	**Regression coefficient (reduction in GI vs GI for maize) (%)**	**Meta-analysis (significant effect of millet-based diet on GI vs. control)**		**Glycaemic index food category**
			Fixed effect model	Random effect model	
Barnyard millet	42.3	−27.2	*P* < 0.01	*P* = 0.02	Low
Fonio	42.0	−28.9	*P* < 0.01	*P* = 0.07	Low
Foxtail millet	54.5	−29.9	*P* < 0.01	*P* < 0.01	Low
Job's tears	54.9	−35.6	*P* < 0.04	*P* = 0.4	Low
Mixed millet	42.7	−26.4	*P* < 0.01	*P* < 0.01	Low
Teff	35.6	−27.1	*P* < 0.01	*P* = 0.31	Low
Finger millet	61.1	−26.0	*P* < 0.01	*P* < 0.01	Intermediate
Kodo millet	65.4	−20.1	*P* < 0.01	*P* = 0.21	Intermediate
Little millet	64.2	−13.3	*P* = 0.98	*P* = 0.31	Intermediate
Pearl millet	56.6	−18.1	*P* < 0.01	*P* < 0.01	Intermediate
Sorghum	61.2	−22.7	*P* < 0.01	*P* < 0.01	Intermediate
**Control**					
Milled rice	71.7	−11.4	NA	NA	High
Refined wheat	74.2	−15.9	NA	NA	High
***In vitro*** **studies**					
Teff	54.3	NA	*P* < 0.01	*P* < 0.01	Low
Fonio	56.3	NA	*P* < 0.01	*P* < 0.17	Low

*P < 0.01, Significantly lower glycaemic index; NA, Not applicable. Fonio and teff data are from a single source; therefore, the results of a fixed effect model were more reliable than a random effect model*.

#### Meta-Analysis

**Figure 2:**
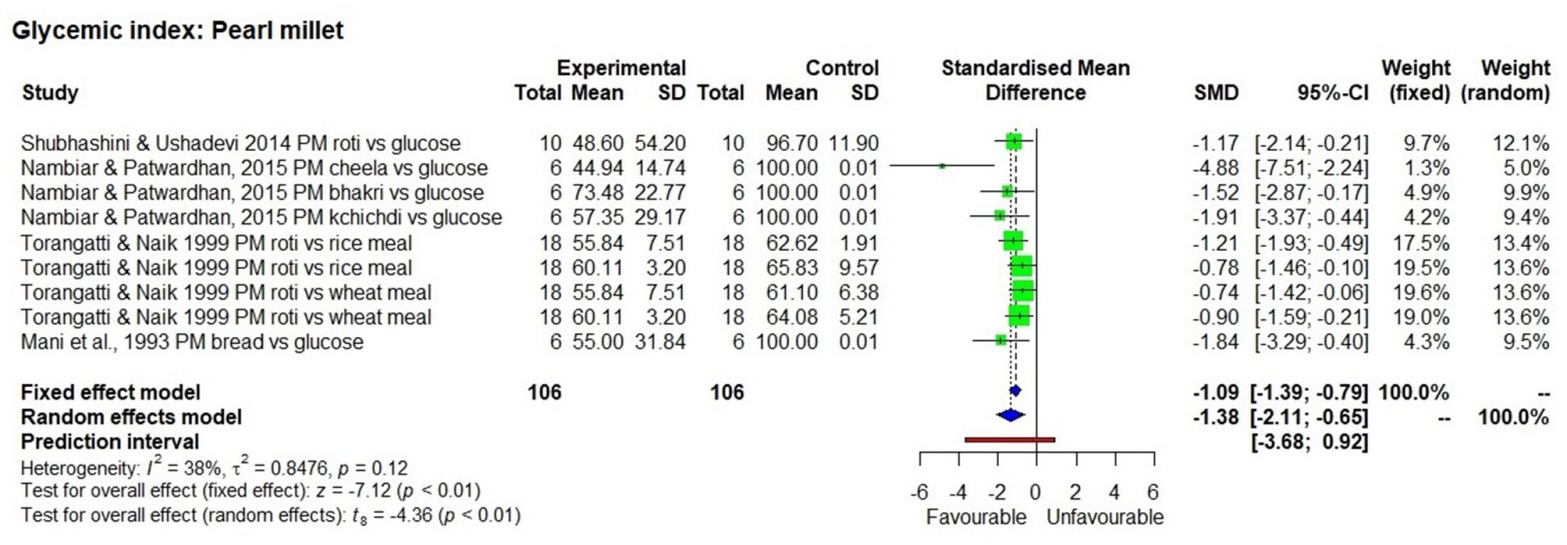
The Glycaemic Index of a pearl millet diet compared to control samples. Abbreviations given in **Figures 2–8:** PM, Pearl millet; FM, Finger millet; DFM, Decorticated finger millet; ER, Expanded rice; WFM, Whole finger millet; GFM, Germinated finger millet; A vs. B, After vs. Before; SMY, Soya milk yoghurt; FMMY, Finger millet milk yoghurt; P, Probiotic; FTM, Foxtail millet; WB, White bread; KM, Kodo millet; KM-SU, Kodo millet sewai upma; RW-SU, Refined wheat sewai upma; SGG, Split green gram; WGG, Whole green gram; MM, Mixed millet; NM, Non-millet; FMFU, Finger millet flakes upma; FMVU, Finger millet vermicelli upma; RSR, Raw small roasted; RSU, Raw small unroasted; RLR, Raw large roasted; RLU, Raw large unroasted; RPSR, Raw parboiled small roasted; RPSU, Raw parboiled small unroasted; RPLR, Raw parboiled large roasted; RPLU, Raw parboiled large unroasted; FME, Finger millet extruded; FMB, Finger millet ball; SGF, Stone ground flour; KM, Kodo millet; MM, Mixed millet; FTM, Foxtail millet; BM, Barnyard millet. Description of food items in **Figures 2–8:** Dosa, Indian pan cake; Roti, a flat round bread cooked on a griddle; Chapatti, a thin flat bread of unleavened wholemeal bread cooked on a griddle; Pittu, Portioned steam cooked cake; Khichdi, Pulse, millet, spices mixed, and cooked together; Laddu, an Indian sweet made from a mixture of flour, sugar, and shortening, which is shaped into a ball; Baati, Hard unleavened bread; Burfi, Indian milk based sweet (here prepared with millet); Upma, a breakfast dish made by simmering roasted grain in tampered and spiced boiling water; Cheela, a savoury pan cake; Bhakri, a round flat unleavened bread.

**Figure 3:**
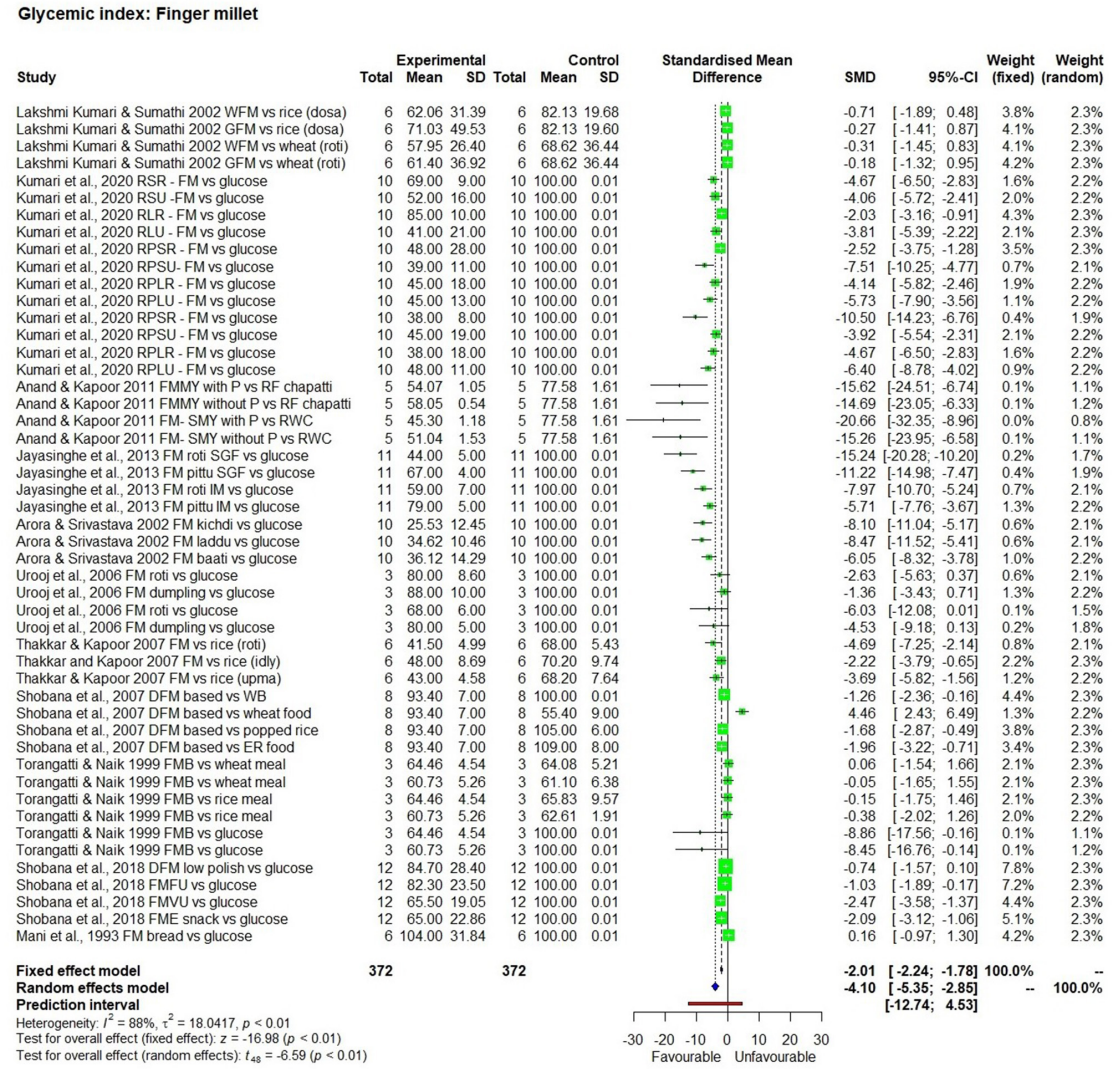
The Glycaemic Index of a finger millet diet compared to control samples.

**Figure 4:**
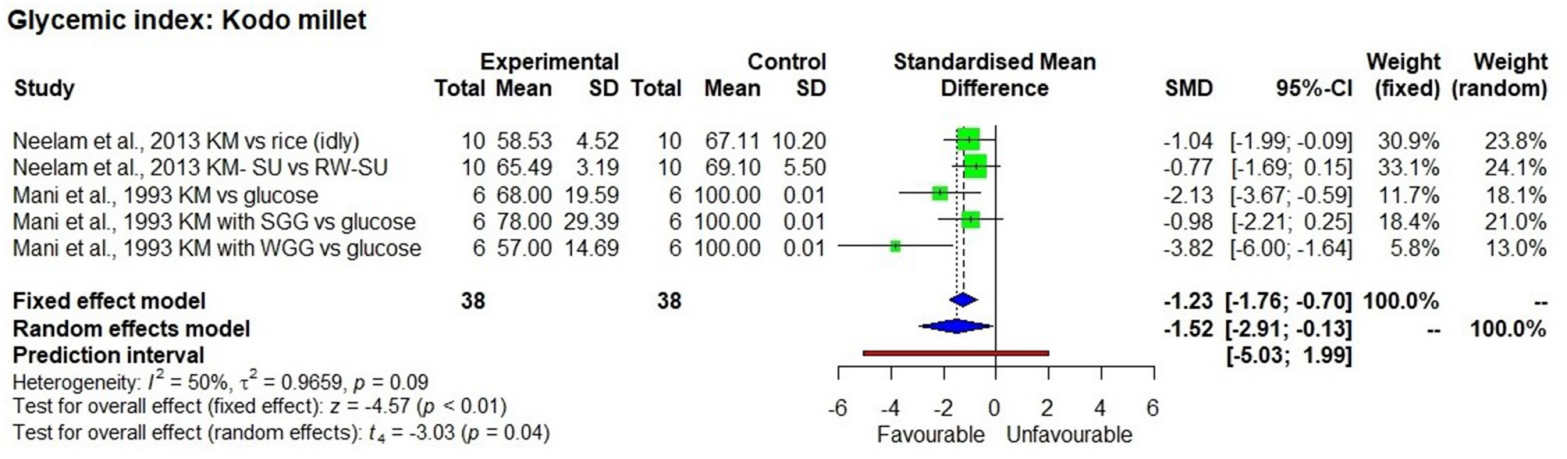
The Glycaemic Index of a kodo millet diet compared to control samples.

The effect of consuming millet-based food compared to the respective control samples or pre-intervention (baseline) values of participants of each study was determined through five outcomes, namely GI value (Figures 2–4), fasting, post-prandial, HbA1c, insulin level of blood in a meta-analysis and a forest plot was generated. The fixed effect model shows that except for little millet, the other 9 millets had a significantly low GI compared to control samples (Table 3). The fixed effect model was useful in explaining that fonio and teff samples were from a single source. Among 11 types of millets and one mixed millet tested, only little millet did not show a significantly lower GI compared with the control samples in both fixed effect and random effect models. There was no single study that determined GI of proso millet therefore it was not used in meta-analysis. All other studies generally showed a significantly lower GI than the control food tested, which included white refined wheat, rice, maize and glucose. Fonio showed low heterogeneity (0%) due to a single source sample and no randomisation with significantly low (*p* < 0.01) GI compared to standard glucose. Little millet had high heterogeneity (97%) with GI which was not significantly low (*p* = 0.31) compared to a rice-based diet. Teff showed moderate heterogeneity (75%) due to a single source sample and less randomisation with significantly low (*p* < 0.01) GI compared to corn injera (a white leavened Ethiopian flat bread with spongy texture) and white wheat bread. Barnyard millet exhibited high heterogeneity (95%) and significantly low GI (*p* = 0.04) with 95% confidence interval of −29.18; −0.99. Sorghum exhibited moderate heterogeneity (75%) and significantly low GI (*p* =0.03) with 95% confidence interval of −2.59; −0.20 with Standardised Mean Difference (SMD) of −1.39. Pearl millet exhibited low heterogeneity (38%) and significantly low GI (*p* < 0.01) with 95% confidence interval of −2.11; −0.65. Kodo millet exhibited low heterogeneity (50%) and significantly low GI (*p* < 0.01) with 95% confidence interval of −1.76; −0.70. Foxtail millet exhibited high heterogeneity (89%) and significantly low GI (*p* < 0.01) with 95% confidence interval of −5.77; −1.44. Finger millet exhibited high heterogeneity (88%) and significantly low GI (*p* < 0.01) with 95% confidence interval of −5.35; −2.85. Mixed millets exhibited high heterogeneity (93%) and significantly low GI (*p* < 0.01) with 95% confidence interval of −10.15; −3.73.

**Table 3:** Heterogeneity and p values from fixed and random effect models from forest plots on glycaemic index, fasting and post-prandial blood glucose levels.

**Millet**	**Heterogeneity (*I^**2**^*) (%)**	**Fixed effect model (*p*)**	**Random effect model (*p*)**	**95%–confidence interval**	**Author details**
**Glycaemic index(GI)**					
Fonio	0	<0.01	0.07	−6,655.5; −3,803.9	(33)
Little millet	97	0.98	0.31	−52.02; 27.43	(8, 34, 35)
Teff	75	<0.01	0.31	−1.98; −0.55	(36)
Job's tears	97	0.04	0.40	0.08; 2.46	(14, 37)
Barnyard millet	95	0.01	0.04	−29.18; −0.99	(8, 38–42)
Sorghum	75	<0.01	<0.01	−2.59; −0.20	(43–46)
Kodo millet	50	<0.01	0.04	−2.91; −0.13	(8, 46, 47)
Mixed millet	93	<0.01	<0.01	−10.15; −3.73	(48–51)
Finger millet	88	<0.01	<0.01	−5.35; −2.85	(41, 44–46, 52–58)
Pearl millet	38	<0.01	<0.01	−2.11; −0.65	(44, 46, 59, 60)
Foxtail millet	89	<0.01	<0.01	−5.77; −1.44	(8, 35, 39, 44, 48, 61–63)
**0 min/fasting blood glucose level**					
Fonio	93	0.80	0.70	22.77; 21.01	(33)
Little millet	0	0.83	0.71	−1.53; 1.42	(8, 64)
Job's tears	87	<0.01	0.77	10.20; 9.64	(37, 65)
Proso millet	51	0.03	0.20	−1.19; 0.34	(66)
Barnyard millet	40	0.04	0.13	−1.19; 0.22	(8, 38, 42, 67)
Pearl millet	0	0.97	0.99	−0.30; 0.31	(8, 44, 46, 60, 68–70)
Sorghum	0	0.49	0.25	−0.31; 0.09	(44–46, 64, 71, 72)
Kodo millet	86	<0.01	0.21	−0.14; 0.32	(8, 46, 68)
Mixed millet	86	<0.01	0.03	−2.48; −0.13	(11, 12, 49, 50, 73–78)
Finger millet	55	<0.01	0.05	−0.52; 0.00	(7, 44, 45, 52–54, 56, 79–81)
Foxtail millet	33	<0.01	0.09	−56; 0.04	(8, 10, 13, 35, 39, 44, 48, 61–63, 68, 82–85)
**120 min/post-prandial blood glucose level**					
Fonio	28	<0.01	0.17	−9.09; 4.98	(33)
Little millet	99	<0.01	0.48	84.88; 88.11	(8, 64)
Proso millet	87	<0.01	0.19	−2.54; 0.70	(66)
Barnyard millet	97	<0.01	0.33	−28.09; 120.33	(8, 38)
Pearl millet	86	<0.01	0.07	−2.89; 0.14	(8, 44, 46, 60, 68–70, 86)
Sorghum	0	<0.01	0.01	−0.82; −0.12	(44–46, 64, 71, 72, 87)
Mixed millet	90	<0.01	0.02	−1.97; −0.27	(49, 50, 73–76)
Finger millet	79	<0.01	<0.01	−3.51; −0.94	(7, 44, 45, 52–54, 56, 64, 80, 81)
Foxtail millet	91	<0.01	0.02	−3.68; −0.29	(8, 9, 13, 44, 61–63, 68, 82)
**Area under the curve glucose**					
Finger and foxtail millet	11	<0.01	0.03	−3.24; −0.23	(88)
Proso millet	37	0.98	0.98	−0.65; 0.66	(66)

#### Fasting and Post-prandial Blood Glucose Level

**Figure 5:**
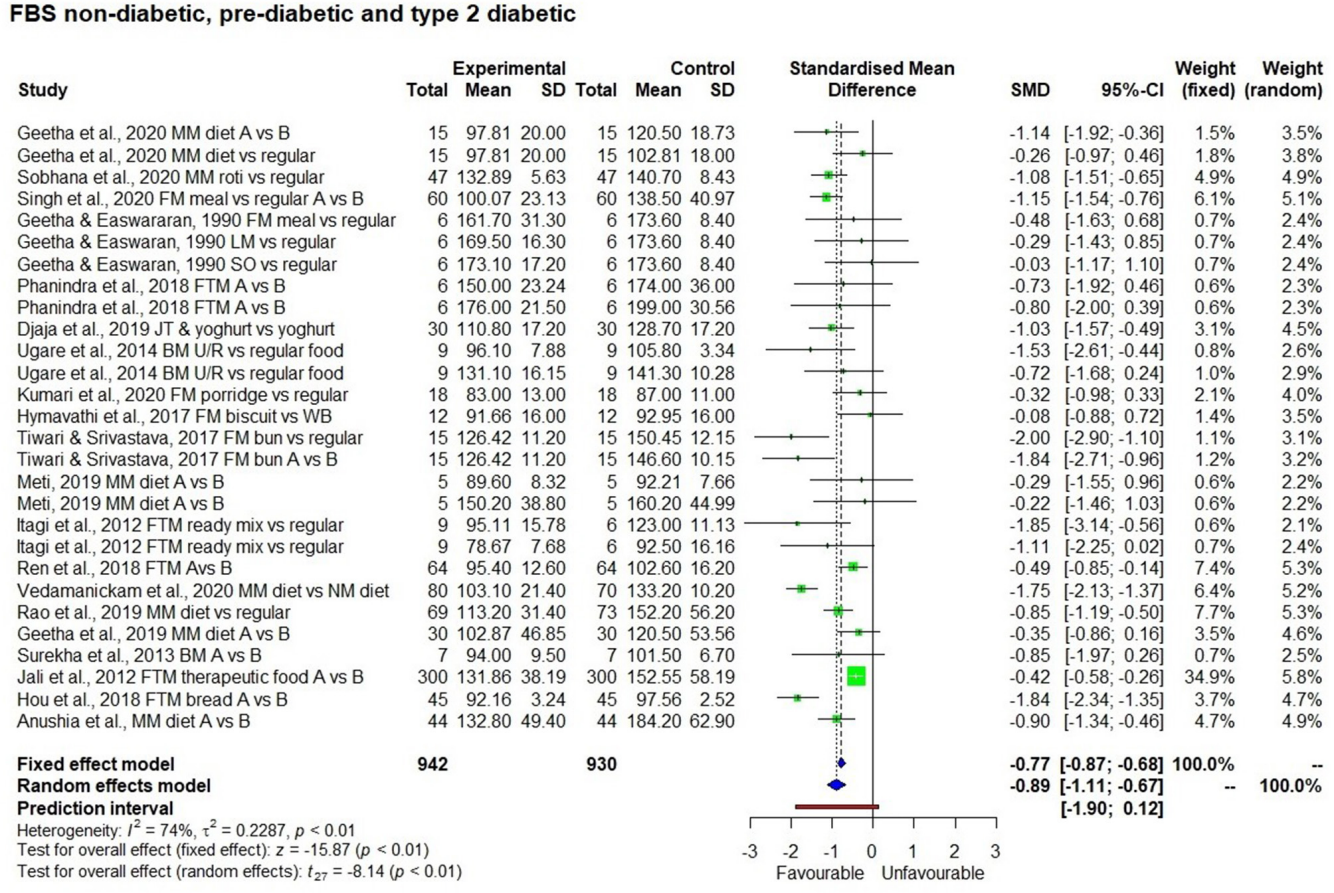
Effect of long term consumption of millet on fasting blood glucose levels in non-diabetic, pre-diabetic, and diabetic subjects compared to the control group consuming a regular diet or pre vs post intervention comparison.

**Figure 6:**
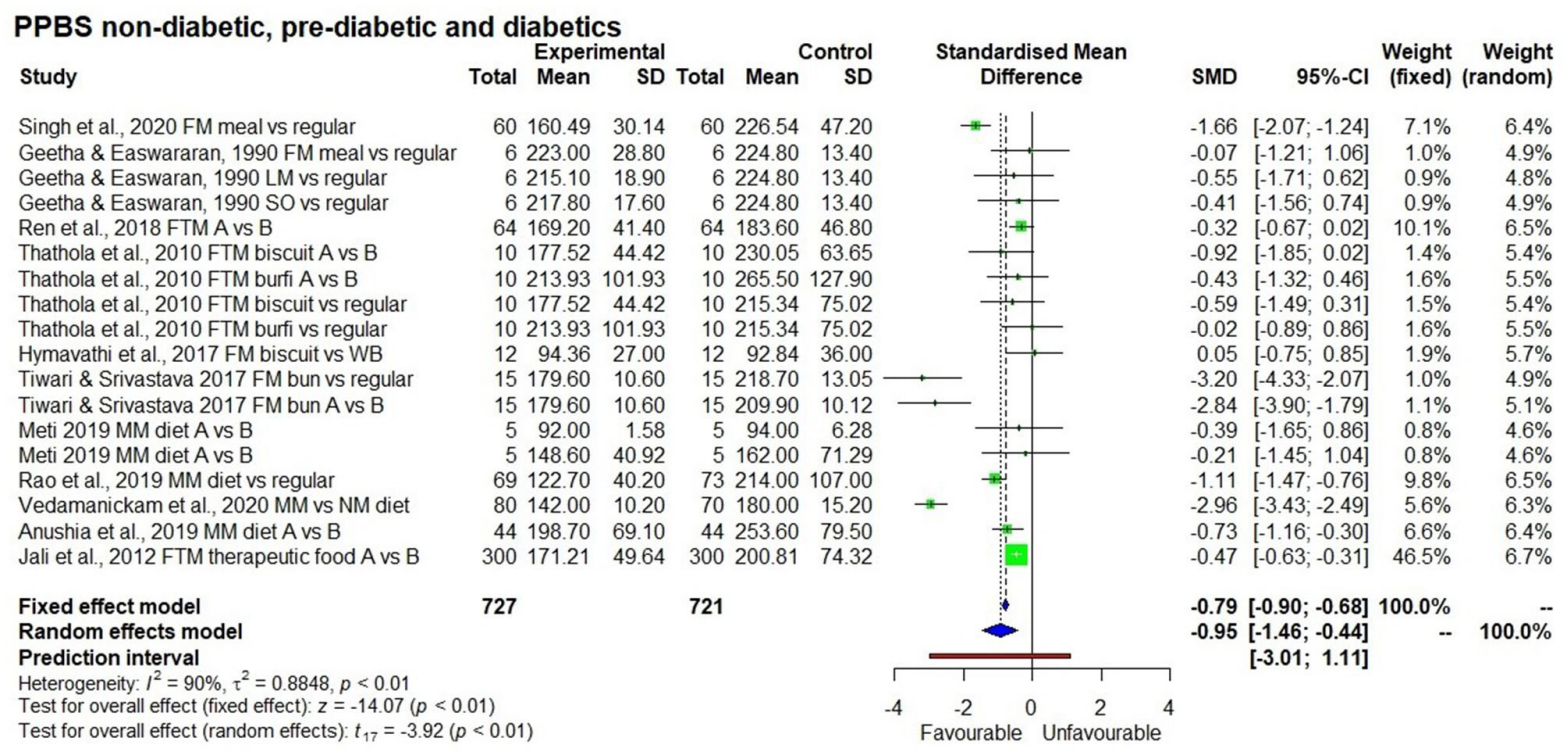
Effect of long term consumption of millet on post-prandial blood glucose levels in non-diabetic, pre-diabetic, and diabetic subjects compared to the control group consuming a regular diet or pre vs post intervention comparison.

In short term studies, all the 9 millets tested for post-prandial blood glucose significantly (Table 3) reduced blood glucose concentration compared to the control sample (*p* < 0.01). However, short term studies with overnight fasting didn't have a significant effect on fasting blood glucose level. In contrast, Figures 5, 6 show the significant effect (*p* < 0.01) being fed on millets for a long time (one study for 7 days and others were for 4 weeks to several weeks) had on reducing fasting (SMD −0.89 with 95% confidence interval of −1.11; −0.67) and post-prandial (SMD −0.95 with 95% confidence interval of −1.46; −0.44) blood glucose levels. While using a random effect model, kodo millet, little millet, and barnyard millet did not have a significant effect on post-prandial blood glucose levels compared to control samples. However, fonio and proso millet came from a single source of reference and the samples were the same; so only a fixed effect model was used in the interpretation which demonstrated a significant effect in reducing post-prandial blood glucose levels.

#### HbA1c

**Figure 7:**
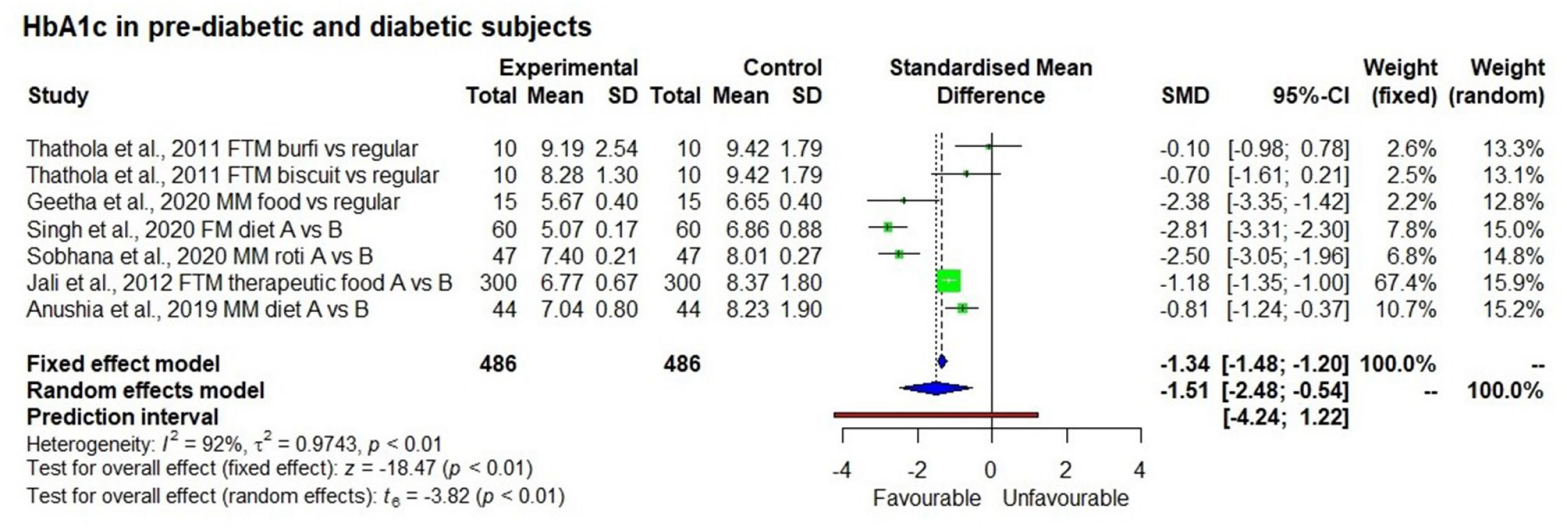
Effect of long term consumption of millet on HbA1c levels in pre-diabetic and diabetic subjects compared to the control group consuming a regular diet or pre vs post intervention comparison.

There were six long term studies conducted to determine the effect of a millet diet on HbA1c level (Figure 7). All of them showed a reduction in HbA1c levels as a result of long term millet consumption; this reduction was significantly lower compared to when consuming a control rice-based diet or pre-intervention (baseline) HbA1c levels (*p* < 0.01).

#### Insulin Level

Albeit not shown, there were five studies that determined the insulin index (1 study with 5 observations), fasting insulin level (2 studies) and Area Under the Curve of Insulin (AUC) (2 studies) as a co-effect of reduction in GI, and the result showed significant reduction in fasting insulin level (*p* < 0.01) and insulin index in fixed effect model with no significant effect on AUC insulin (*p* = 0.24).

#### Subgroup Analysis

**Figure 8:**
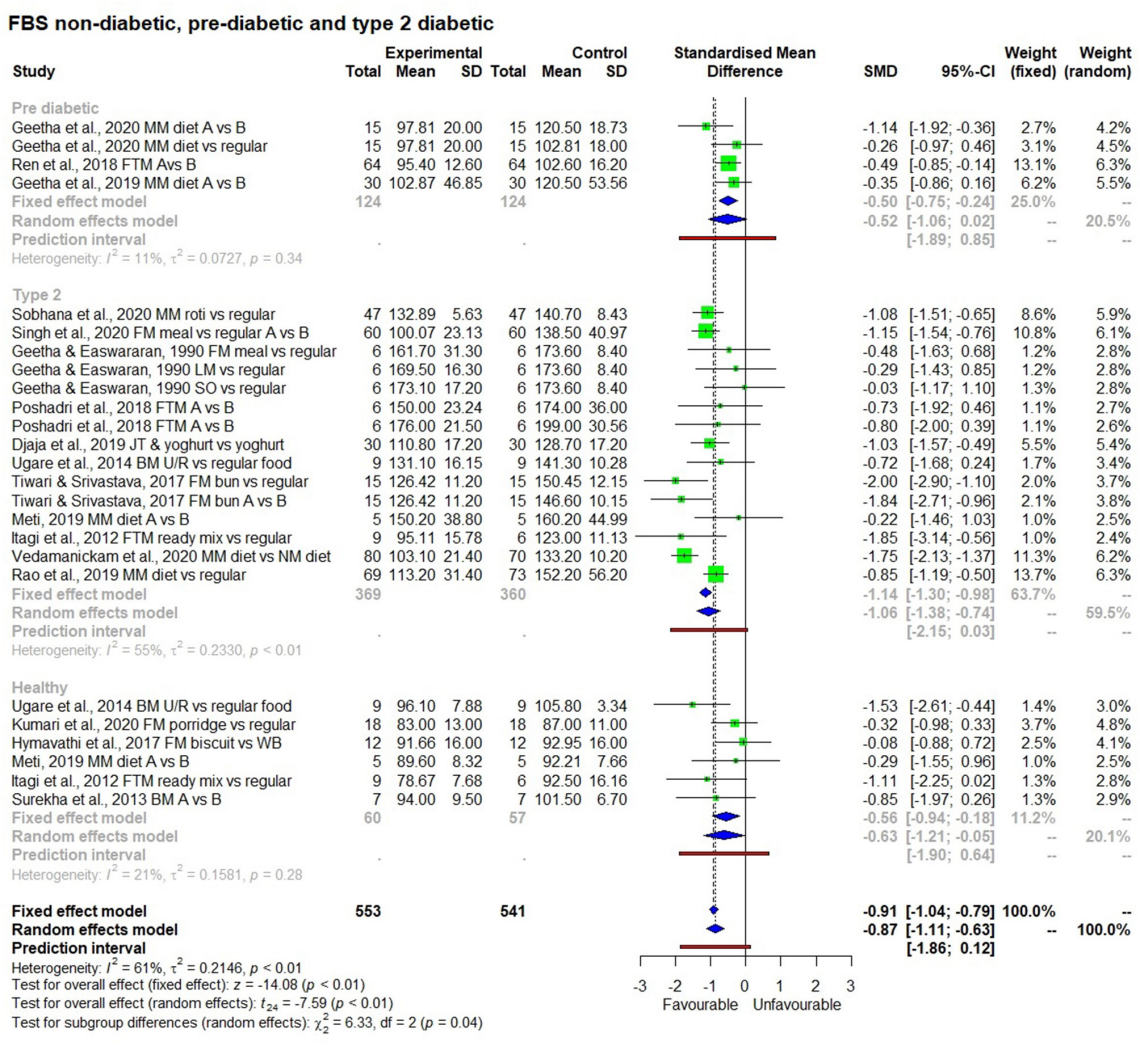
The effect of long term consumption of millet on non-diabetic, pre-diabetic and diabetic subjects consuming millet compared to a control group consuming a regular diet.

Results of the subgroup analysis (Figure 8) showed that consuming a millet-based diet for a long time (>3 months) had a significant effect on reducing fasting blood glucose levels in all participants regardless of the group (non-diabetic, pre-diabetic, and diabetic) compared to a regular rice or wheat-based diet (*p* < 0.01). There was no significant difference among groups (*p* < 0.13). However, when looking at post-prandial blood glucose level, a significant reduction in blood glucose was observed among type 2 diabetic subjects compared to non-diabetic ones and the subgroup effect was significant (*p* < 0.01). It was not possible to see this difference between diabetic and pre-diabetic subjects due to the small number of studies on the latter. The subgroup effect was not significant (*p* = 0.69) based on the type of millet in reducing both fasting and post-prandial blood glucose levels; this goes to show that regardless of the type of millet, its long term consumption has the potential to reduce both fasting and post-prandial blood glucose levels.

### Regression Analysis

GI levels varied among various millets with the average GI of low to intermediate. Various millets and food forms tested in 63 studies with 267 observations on millets and sorghum and 267 observations on maize, wheat, rice, or reference food (glucose or wheat bread). Millets had low GI (≤55%), lower than milled rice, refined wheat flour, white wheat bread, maize, or glucose (reference food).

Table 4 presents the frequency distribution of all the categorical independent factors included in the regression analysis. Finger millet and foxtail millet were the most frequently observed crops, followed by rice, wheat and sorghum. Most cases used food made from a refined grain, while a few cases used food originating from whole grain. Boiling was the most common cooking method, followed closely by baking (and/or roasting). About 14% of the cases used the *in vitro* estimation of GI values while the rest tested on normal subjects (59%) and type 2 diabetic subjects (27%).

**Table 4:** Frequency distribution of the independent variables in the regression analysis: crop, cooking method, and method used to determine GI (*n* = 267).

**Crop**	**Number of observation**	**%**
° Job's tears	2.0	0.7
° Barnyard millet	16.0	6.0
° Finger millet	46.0	16.9
° Fonio	7.0	2.6
° Foxtail millet	33.0	12.4
° Kodo millet	10.0	3.8
° Little millet	3.0	1.1
° Pearl millet	11.0	4.1
° Mixed millet	21.0	7.9
° Sorghum	18.0	6.7
° Teff	3.0	1.1
° Maize	11.0	4.0
° Rice	32.0	12.0
° Refined wheat	26.0	9.7
° Others	15.0	5.6
° Roots and tubers	3	1.1
° Legumes	3	1.1
**Cooking method**		
° Bake and/or roast	119.0	44.6
° Boil	119.0	44.6
° Steam	17.0	6.4
° Raw	12.0	4.5
**Method used to determine GI**		
°*In vitro*	38.0	14.0
° Human testing on normal subjects	157.0	58.8
° Human testing on type 2 diabetes subjects	72.0	27.0

Table 5 shows the results of the Cluster-Robust OLS analysis of the effects of different crops on GI values. To keep the table succinct, the 63 control variables included controlling for literature fixed effects were dispensed with. The result indicates that 14 out of the 18 analysed crops had negative and statistically significantly lower (*p* < 0.10) GI values compared to maize flour-based control food. In descending order, the marginal effects were −35.6 for Job's tears, −28.9 for fonio, −29.9 for foxtail millet, −27.1 for teff, −27.2 for barnyard millet, −26.4 for mixed millet, −26.0 for finger millet, −22.7 for sorghum, and −20.1 for kodo millet.

**Table 5:** The effects of crop choice on GI values compared with maize, using ordinary least squares with cluster-robust standard errors.

	**Coef**.	**Robust SE**	***p*-value**
**Crop**			
Job's tears	−35.580^***^	12.620	0.006
Barnyard millet	−27.168^**^	11.633	0.023
Finger millet	−26.012^*^	13.186	0.053
Fonio	−28.900^**^	10.933	0.010
Foxtail millet	−29.858^**^	11.662	0.013
Kodo millet	−20.068^*^	11.235	0.079
Little millet	−13.336	17.356	0.445
Pearl millet	−18.064	11.696	0.127
Mixed millet	−26.426^**^	10.941	0.019
Sorghum	−22.657^*^	12.267	0.069
Teff	−27.096^**^	10.534	0.012
Rice	−11.448	12.439	0.361
Refined wheat	−15.882	11.265	0.164
Wheat based	−37.826^***^	10.941	0.001
Legumes	−37.006^*^	21.804	0.095
Others	−21.719^**^	10.008	0.034
**Cooking method**			
Baked and/or roasted	16.361^***^	3.382	0.000
Boiled	11.329^***^	2.491	0.000
Steamed	18.405^***^	4.537	0.000
**GI estimation methods (base: human testing on normal subjects)**			
Human testing on type 2 diabetes subjects	5.275^***^	1.598	0.002
*In vitro*	−24.928	6.397	0.644
Constant	71.663^***^	6.778	0.000

*Dependent variable = Glycaemic Index (GI) value: n = 267, R^2^ = 0.660, Adj. R^2^ = 0.416*.

*NB, The estimation included 63 literature dummies which were not included in the table*.

*** *p < 0.01*,

** *p < 0.05, and*

* *p < 0.10, respectively*.

## Discussion

Most of the studies showed a glucose-lowering effect of various types of millets that were served in various forms compared to the control foods. A variety of processed products and cooking methods were tested and often compared to milled rice, refined wheat and maize-based foods. The regression analysis clearly shows that millets have a lower GI compared to other cereals such as maize, milled rice and refined wheat flour. This means, for instance, that when Job's tears-based food was consumed, the GI value was significantly lower by 36 units on average than when maize-based food was consumed, taking into account that all the other conditions (i.e., processing, cooking methods, type 2 diabetes condition, and GI estimation methods) were equal. Similarly, when foxtail millet-based food was consumed, the GI value was significantly lower by 30% on average than maize-based food (Table 5). It may be noted that Job's tears-based food is comparable with whole wheat-based food and legumes as these two foods lower GI by 37.8 units and 37.0 units, respectively on average than the consumption of maize-based food. Major crops such as milled rice and refined wheat did not show a GI advantage against maize, indicating that they tend to have relatively high GI values. On the other hand, among the broad group of millet crops (millets, sorghum, and teff), all of them showed lower GI values except little and pearl millet, for which the coefficient was negative (−13.3 and −18.1) but not statistically significant (*p* = 0.445 and 0.127).

All the cooking methods raised GI values. In particular, steaming, baking (including flat bread cooked in a pan) and boiling increased the GI of the food by up to 18.4 units, 16.3 units and 11.3 units, respectively. Despite this, the overall GI of millets was 52.7. This could be due to the addition of other ingredients such as fats and oils in different types of cooking. Somewhat unexpectedly, the use of whole grain millets did not affect GI values significantly compared to decorticated millets. This could be because of the fewer sample numbers that used whole grain.

The coefficient of type 2 diabetes showed that subjects with type 2 diabetes tend to exhibit higher GI (+5.3, *p* = 0.002) values after a meal compared to those without diabetes. The coefficient of the *in vitro* estimation was not significant, implying that on an average the GI values were not different when *in vitro* estimation was used instead of human testing on non-diabetic subjects, which supports the validity of the GI values estimated with *in vitro* experiments. Although not included in the table, the additional analysis using the interaction terms between the type 2 diabetes condition and crop variables showed that the GI benefits from millets such as barnyard millet, finger millet, fonio, foxtail millet, kodo millet, pearl millet, and sorghum did not differ between type 2 diabetic subjects and non-diabetic subjects. This indicated that these millets may be more effective in lowering GI values compared to major cereals irrespective of whether the subjects were diabetic or not. These findings demonstrate that the consumption of food items made from various millets contribute to keeping the blood glucose level low compared to the food based on maize and milled rice. Moreover, barnyard millet, fonio, foxtail millet, kodo millet, pearl millet, and sorghum were equally beneficial for type 2 diabetes and non-diabetes individuals.

The regression analysis' results were generally supported by the meta-analysis conducted for the data on GI which showed that all the studies except those on little millet had no significant effect on reducing blood glucose levels.

Two forest plots constructed during the meta-analysis were repeated in different ways to determine the effect of removing one study that was identified as an outlier or having an odd Standard Mean Difference (SMD) value. In finger millet, the study conducted by Ruhembe et al. (89) showed highest SMD of 230 while the overall SMD of the study was −1.84. Removing this particular study changed the overall effect with an SMD of −3.38. Similarly in sorghum, the same study showed highest SMD of 311.16 vs. an overall SMD of 14.49; removing the study changed the SMD to −1.2 and the *p*-value became more significant. These two studies were masking the effect of other studies, and this could be because of the lack of non-random sample selection and allocation, no blinding test (both participants and the testing person) and eventually scored highly critical rank in risk assessment. Therefore, the risk of bias could be reflected in getting small standard deviation between sample and high SMD in the meta-analysis. There were only 5 studies available on little millet, of which 2 didn't have complete data on SD and hence were not used in the meta-analysis. Of the 5 studies, only 1 reported that little millet has high GI (35). The SMD value reported by Malavika et al. (35) deviated highly from all other studies. If that one study was removed, then little millet showed a significant effect on reducing GI in the fixed effect model (*p* < 0.01). The high GI in little millet was attributed to polishing millets. However, this study needs a detailed evaluation to generate more evidence on little millet given the limited number of studies available. Proso millet was studied by only one author (66) who didn't calculate GI but studied the change in blood glucose level for a period of 2 h after the consumption of proso millet products which showed significant reduction in blood glucose level (*p* < 0.05).

It may be noted that consuming a millet-based diet for long periods (more than 3 months) was also associated significantly with reduced HbA1c marker levels in both pre-diabetic and diabetic subjects (*p* < 0.01) compared to consuming a regular rice or wheat-based diet or pre-intervention HbA1c level. HbA1c is a glycated haemoglobin, i.e., it is bound to glucose and is different from free unbound glucose in blood. Unlike fasting blood glucose level which reflects the blood glucose level at a particular point of testing time, HbA1c reflects the average blood glucose level typically over a period of 8 to 12 weeks and is therefore an indicator of long-term glycaemic control. Overall, there was a 15% reduction in HbA1c level (from 8.1 ± 1.0 to 7.0 ± 1.4%). Especially in pre-diabetic subjects, HbA1c levels fell to the normal reference level (from 6.65 ± 0.4 to 5.67 ± 0.4%) (12). The reduction is attributed to the high fibre content and low glycaemic index of the millet-based diet (11) which reduces the availability of glucose to form HbA1c and thereby regulates the HbA1c glycation process. It is evident that a millet- based diet has a positive effect on managing diabetes.

Another study conducted on pre-diabetic subjects (those with impaired glucose tolerance) fed on foxtail millet (82) for a long period (12 weeks) showed that the fasting blood glucose level reduced to normal levels (from 102.6 ± 16.2 to 95.4 ± 12.6 mg/dl) in 64 study subjects (*p* < 0.001). This is evidence of millets' effect on averting rising blood glucose levels and preventing pre-diabetic individuals from entering the diabetic stage. However, more studies are needed to reconfirm this.

It is important to note that most of the studies were conducted after overnight fasting and the introduction of the test food or control food as breakfast. This was followed by the measurement of fasting and post-prandial blood glucose levels. This method does not give information on how the glycaemic response might change after acclimatisation to millet-based food. However, 21 studies conducted using millet as a test food for long periods of time ranging from 7 days to several weeks after which fasting and post-prandial blood glucose levels were measured, provided information on changes in both levels after acclimatisation to millet-based food. The results show that consuming millet for a long duration has a positive effect of reducing both fasting blood glucose level (*p* < 0.05) by 12%, with a mean reduction of 16 mg/dl (from 134 mg/dl to 117.9 mg/dl) and post-prandial blood glucose level by 15%, with a mean reduction of 30 mg/dl (from 202 to 172 mg/dl) which is near normal levels for diabetic subjects. While testing after overnight fasting (short term studies) had no significant effect on fasting blood glucose level, there was a significant reduction (*p* < 0.05) in post-prandial blood glucose level.

There were only two studies (14, 62) that determined insulin index and GI. It may be noted that although Job's tears' GI was low (55), its insulin index was slightly higher (67). The insulin index in Job's tears was less compared to brown rice (81%) and Taro or colacasia esculenta, a root vegetable (73%). The author of these studies ascribed the insulin response of the food increase to the co-injection of protein or fat through the meal. This clearly suggests the need for extreme caution while preparing food for diabetic individuals to ensure it has not just low GI but also a low insulin index to avoid raising insulin levels in the blood; high insulin concentration is associated with insulin resistance and cardiac risk (14). Consuming millet based diet for three months was shown to increase in mean insulin sensitivity from 68.1 ± 4.7 to 88.2 ± 6.0 (11). Ren et al. (62) demonstrated that when foxtail millet was cooked with only water, the insulin index was very low (49.8) compared to processed food, and the ratio of insulin index and GI was <1 compared to the processed products. Hence, it was reported as a suitable product for managing diabetes.

Several studies have shown that resistance starch formation in millets and high fibre in millet retard starch hydrolysis, thereby exhibiting low GI (45) and its potential to reduce blood glucose level. The high presence of a non-starch polysaccharide such as dietary fibre in millets compared to wheat and rice (90) decreases enzymes' activities in the gut and results in incomplete hydrolysis of carbohydrates, protein and fats present in millet-based diets. This delays the absorption of starchy polysaccharides and lowers the rate of absorption of mono and disaccharides (46), thereby exhibiting low glycaemic response. High resistant starch formation in millets is due to the presence of amylose which tends to retrogradation of starch (set back viscosity) which forms resistant starch and thereby is difficult to hydrolyze by digestive enzymes (61), leading to low glycaemic response. Also, fat and protein content in any food slow down the rate of gastric emptying, thereby slowing down the digestion of food in the intestine. Millets are known to have high protein and fat compared to milled rice (90) and thereby contributing to low GI (61), this is because, protein and fat combined with other factors slows down the digestion in small intestine which leads to incomplete digestion and thereby contributes to low GI. Protein content in millet increases insulin sensitivity thereby helping to maintain better glycaemic response.

Lakshmi Kumari and Sumathi (52) and Abdelgadir et al. (87) reported that high fibre content in finger millet gives rise to slower gastric emptying or the formation of non-absorbable complexes with carbohydrates in the gut lumen. Itagi et al. (10), Thilakavathy and Muthuselvi (68), Pathak et al. (48) and Narayanan et al. (13) have also reported the glucose lowering effect of finger millet due to high-soluble dietary fibre in food which reduces gastric emptying, the absorption of glucose after a meal and decreases the activity of digestive enzymes. This results in incomplete hydrolysis of carbohydrates, protein and fats, thereby delaying absorption. Jayasinghe et al. (55) reported that when two different processing methods such as stone milling and industrial milling were used to make flour, the large particles of flour produced make starch gelatinization relatively difficult and slow down enzyme attack. This slows down the release of glucose from food, causing a significant decrease in glycaemic response. Nambiar and Patwardhan (60) reported both high GI of some foods and low GI of others which they attribute to processes like boiling and pressure (steam) cooking that result in faster rates of digestion compared to roasting. This could be the reason for the high GI in *khichadi* (a mix of pulse, millet, spices) compared to *cheela* (savoury pancake)*, thalipeeth* (savoury multi-grain flat bread), sorghum *bhakri* (round flat unleavened bread), and wheat roti. It is further confirmed in current systematic review, that boiling of millet in whole or decorticated form either unprocessed or minimally processed by milling into coarse grain or flour produced average GI of 52.1 ± 3.9 (low GI) compared to milled rice (63.1 ± 10.7) or maize (58.8 ± 18.9). In addition, Ren et al. (82) clearly demonstrates that including foxtail millet in the diet can reduce fasting blood glucose level provided the consumer is restricted to the specified diet, which is important contributing factor in achieving impact.

**Figure 9:**
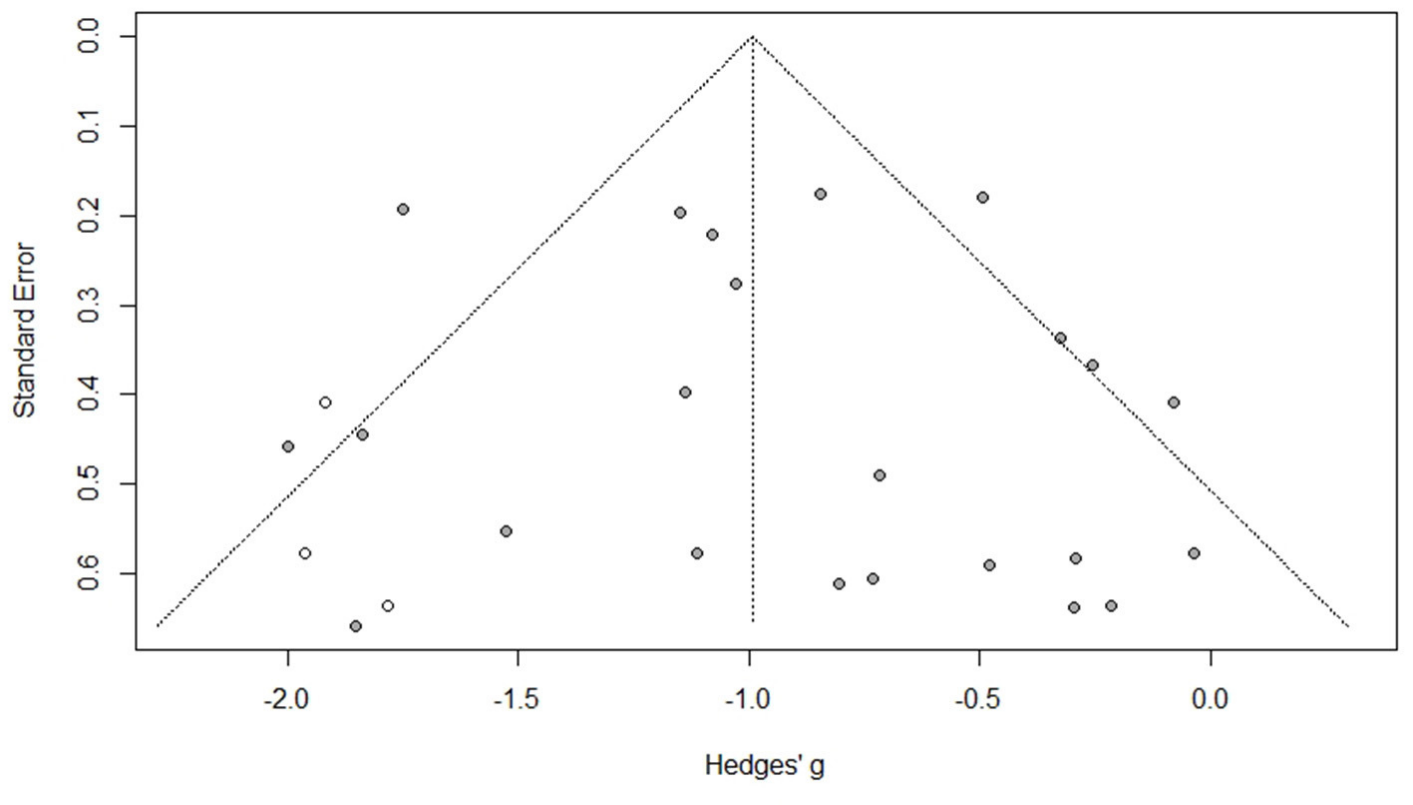
Publication bias assessment of the long term studies used for fasting blood glucose level as an effect of consuming millet-based meal (after applying trim and fill method) (*p* < 0.0001).

A risk of bias assessment conducted on all the 65 studies revealed that more than 50% of them had low risk of bias. High risk of bias in the overall effect is contributed by blinding of samples tested. Some studies indicated that blinding was not possible with millet-based foods due to their unique texture, flavour and appearance (66, 82). However, participants were blinded for the proportion of millet in any food tested and the name of the millet (70, 86). The asymmetrical funnel plot obtained was due to the small sample size which created publication bias. This effect on the funnel plot was adjusted and accounted for using trim and fill method until the plot became symmetrical (*p* < 0.0001; Figure 9).

### Limitations of the Study

Most of the *in vivo* studies included in the systematic review did not have the standard number of 8–12 subjects to determine GI, as recommended by FAO/WHO (91). Some studies had as few as three subjects, which is a major limitation; but they were not excluded considering the limited number of studies available for some of the millets and the importance of this information. There were only two studies conducted on pre-diabetic subjects to establish the link between millet-based food and its diabetes preventing effect through the reduction of HbA1c and fasting blood glucose levels from higher to normal range. The great variability in using control food further reduced the sample size corresponding to each control. The age group effect was not analysed as most of the studies presented the age group as mean age group rather than the range, which was another limitation.

### Recommendation on Methodology for Future Research

The study captured evidence from the 1990s to 2020. There was no uniformity in method used; only a few studies mentioned having followed 2010 ISO standards. Using this standard to determine GI improves the accuracy of results and uniformity among different studies (regardless of geography and laboratory) as the standard deviation obtained from different laboratories using ISO 2010 is much lower (21).

It may perhaps be helpful to conduct interventions of longer duration by using the continuous glucose monitoring system (92). This system with a sensor can analyze interstitial fluid glucose levels at 15-min intervals for 24 h for 14 consecutive days. It can calculate the mean 24 h interstitial glucose values and incremental area under the curve (iAUC) over the 14 days for an intervention diet and the control diet and iAUC for an individual meal. The use of this system is recommended in future interventions to enhance the accuracy of results in order to generate robust and better evidence on glucose management using millets.

## Conclusions

This systematic review and meta-analysis confirm that the millets evaluated have strong potential in dietary management and the prevention of diabetes. Apart from policy implications, it has implications in terms of nutrition sensitive agriculutre interventions with millets and sorghum and on the dissemination of the beneficial effect of millets and sorghum for glycaemic control.

## Data Availability Statement

The original contributions generated for the study are included in the article/Supplementary Material, further inquiries can be directed to the corresponding author/s.

## Author Contributions

SA and JK-P: conceptualisation. SA, JK-P, and KDVP: review and selection of papers. SA, DIG, RKB, AR, TWT, and MV: writing. JK-P: resource. SA, KS, DJP, KDVP, AR, and MV: data collection, screening, and extraction. SA, RB, and TWT: data extraction, meta-analysis, regression analysis, and risk assessment. SA, JK-P, KDVP, DIG, AR, DJP, KS, RKB, and MV: review and writing. All authors contributed to the article and approved the submitted version.

## Conflict of Interest

The authors declare that the research was conducted in the absence of any commercial or financial relationships that could be construed as a potential conflict of interest.
