# Supplementary material for: A Systematic Review and Meta-Analysis of the Potential of Millets for Managing and Reducing the Risk of Developing Diabetes Mellitus
Source: Front Nutr. 2021 Jul 28;8:687428. doi: 10.3389/fnut.2021.687428 (PMC8355360; doi:10.3389/fnut.2021.687428)
Supplement: Supplementary file 1 [file Table_1.docx]

Table 1. Studies used in meta-analysis of GI, fasting and post prandial blood glucose, insulin index and HbA1c.

| **Author** | **type of grain used** | **Country** | **product (local name)** | **Condition of the participant, sample size (n) and age group** | **Study Design** | **Parameters studied** |
| --- | --- | --- | --- | --- | --- | --- |
| Abdelgadir et al., 2004 | Sorghum | Sudan | Flat bread, porridge | Diabetic participants (n=10), age (50.2 ± 5.3 yrs) | Cross over study in which the participants were fed with sorghum, maize and wheat based food in random order. | AUC insulin, AUC glucose, post prandial glucose |
| Alegbejo et al., 2011 | Fonio | Nigeria | Acha meal | Diabetic individuals (n=10), age (50.10 ± 2.7 yrs); healthy participants (n=7), age (53.50 ± 2.8 yrs) | Controlled trial | GI, fasting and post prandial glucose |
| Aliyu et al., 2020 | Pearl millet | Nigeria | Dambu | Healthy participants (n=20), age not mentioned | Cross over study in which participants were fed with pearl millet and maize dambu | GI, fasting and post prandial glucose |
| Alyami et al., 2017 | Finger and Pearl millet | UK | Porridge | Healthy participants (n=7), age (20.9 ± 0.9 yrs) | Randomized four way cross over study | IAUC glycaemic response (2hrs) |
| Alyami et al., 2019 | Pearl millet | UK | Porridge | Healthy participants (n=26), age (28.5 ± 9.6 yrs) | Randomized two way cross over study | Fasting glucose, IAUC glucose and insulin (2hrs) |
| Anand and Kapoor, 2011 | Finger millet | India | Chapatti | Diabetic participants (n=15), age (50 to 55 yrs) | Self-controlled trial | fasting and post prandial glucose |
| Anju and Sarita, 2010 | Foxtail, Barnyard millet | India | Biscuits | Healthy participants (n=13), age (22 to 27 yrs), | Cross over study. | GI |
| Anushia et al., 2019 | Millet | India | Meal | Type 2 diabetes participants n=88, age (30 to 45 yrs) | Feeding millet after diet counselling to intervention group, control group didn’t receive any counselling and were consuming regular diet | HbA1c, Fasting and post prandial blood glucose level |
| Arora and Srivastava, 2002 | Finger millet, barnyard millet | India | Kchichdi, Laddu, baati | Healthy participants n=10, age (20 to 27 yrs) | Cross over study | GI |
| Bisht and Srivastava, 2013 | Foxtail, barnyard millet | India | Burfi | Diabetic (n=10), age (22 to 27 yrs) | Cross over study | GI |
| Chhavi and Sarita, 2012 | Foxtail millet, finger millet | India | Bread | Healthy (n=10), age (24 to 27 yrs) | cross control study | AUC glucose |
| Dereje et al., 2019 | Teff | Ethiopia | Injera | Healthy (n=10), Age 23 ± 1.6 | Cross over study. Corn injera is control and white bread is reference | GI, fasting and post prandial blood glucose |
| Djaja et al., 2018 | Job’s tears | Indonesia | Job’s tear rice with yoghurt | Diabetic (n=30), control (n=30), age (30 to 60 yrs), | Randomised case control trial conducted for long term (12 weeks) Intervention fed with job tears mixed with yoghurt; control fed with only yoghurt | fasting blood glucose |
| Geetha and Easwaran, 1990 | Sorghum, finger, little millet | India | Idly, dosa, chapatti, sewai and kozhukattai | Diabetic participants (n=30), age (40 to 60 yrs) | Long term feeding intervention (30 days) | Fasting and post prandial blood glucose |
| Geetha et al., 2019 | Mixed millet | India | dumpling | Healthy, intervention (n=30), control (n=30) | Long term feeding intervention (120 days) | Fasting blood glucose |
| Geetha et al., 2020 | Mixed millet | India | dosa, roti, dumpling | Healthy participants (n=10), age (18 to 30 yrs) | Long term feeding intervention (120 days) | GI, fasting blood glucose, HbA1c level |
| Gowri manohari and Poongodi vijayakumar 2013 | Mixed millet | India | Ready to cook prebiotic food | Heathy participants (n=15), age (20 to 25 yrs) |  | GI |
| Hou et al., 2018 |  |  |  |  |  |  |
| Hymavathi et al., 2017 | Finger millet | India | Biscuit | Healthy (n=12), age (20 to 50 yrs) | Long term feeding intervention (21 days) | GI, fasting and post prandial blood glucose |
| Itagi et al., 2012 | Foxtail millet | India | rice, upuma, pancake or thalipattu | Diabetic, intervention (n=9), control (n=6), Age 50.78 (mean) for intervention, 51 (mean) for control group | Long term case control study (4 weeks), by feeding foxtail millet rice, upuma, pancake or thalipattu | GI, fasting blood glucose |
| Jali et al., | Foxtail millet | India | Therapeutic food | (n=300) | Randomised cross over study | Fasting, postprondial, HbA1c |
| Jayasinghe et al., 2013 | Finger millet | Srilanka | Roti | Healthy (n=10), age (20 to 27 yrs) | Cross over study | GI |
| Joshi and Srivastava, 2013 | Barnyard millet | India | Khichdi | Healthy participants (n=10), age (24 to 26 yrs) | Cross over study | GI, fasting and post prandial glucose |
| Kavita and Prema, 1997 | Finger millet | India | Meal | Diabetic (n=20), age 40 to 50 yrs | Cross over study | Fasting and post prandial blood glucose |
| Kumari et al., 2020 | finger miller |  | porridge | Intervention (n=10), Age 24.5 ± 1.33, normal healthy participants |  | GI |
| Lakshmi and Vimla, 1996 | Sorghum | India | Missiroti, upma, dhokla | Diabetic (n=6), age (45 to 60 yrs) | cross over study in which whole and dehulled sorghum was used as test meal and wheat was used as control meal. | GI, fasting and post prandial blood glucose |
| Lakshmi Kumari and Sumathi, 2002 | Whole and germinated finger millet | India | Dosa, roti | Diabetic (n=6), age (40 to 55 yrs) | Cross over study | GI, fasting and post prandial blood glucose |
| Lestari et al., 2017 | Foxtail millet (15%) | Indonesia | cookies | Intervention (n=12), Age (21 to 22 yrs), normal healthy participants | clinical observation – glucose given as food reference. Test food (foxtail millet cookies bar given after one week) | GI |
| Lin et al., 2010 | Job’s Tears (Adlay) | China | meal | Healthy (n=10+10), age (20 to 30 yrs) | Cross over study | GI, Insulin index |
| Malavika et al., 2020 | Little, foxtail millet | India | Cooked like rice | Healthy participants (n=12), age (20 to 45 yrs) | Cross over study | GI, fasting and post prandial blood glucose |
| Mani et al., 1993 | Sorghum, Kodo, Kodo with split green gram, Kodo with whole green gram, pearl, finger millet | India | roasted bread | Diabetic participants (n=6), age (63 ± 12) | Cross over study. | GI, fasting and post prandial blood glucose |
| McSweeney et al., 2017 | Proso millet | Canada | couscous, extruded snack, porridge, biscuits | intervention (n=12), age 26.3 ± 3.8, healthy normal participants | Cross over study | AUC glucose, fasting and post prandial blood glucose level. |
| Meti, 2019 | Mixed millet | India | Multi millet mix | Diabetic participants (n=5), healthy participants (n=5) | case control study with feeding for 7 days | Fasting and post prandial blood glucose |
| Nagaraju et al., 2020 | Mixed millet | India | Roti | Healthy participants (n=12), age (21 to 40 yrs) | Cross over study | GI |
| Nambiar and Patwardhan, 2015 | Pearl millet | India | Cheela, bhakri, Kchidi, | Healthy participants (n=6), age group (18 to 21 yrs) | Cross over study | GI |
| Narayanan et al., 2016 | Foxtail millet | India | Dosa | Diabetic (n=105), age 49.3 ± 9.9 yrs | Cross over study. Day 1 – 52 had millet dosa, day 4 – 53 had rice dosa (control) | GI, fasting and post prandial blood glucose |
| Neelam et al., 2013 | Kodo millet | India | Idly, sewai upma | Healthy (n=10), age (20 to 25 yrs) | Cross over study design The same subjects were fed with different test and control (rice based) fed on different days. | GI, IAUC |
| Nidhi et al., 2014 | Mixed millet | India | Cheela, uthapam | Healthy (n=20), age (20 to 25 yrs) | Cross over study | GI |
| Palanisamy and Sree, 2020 | Kodo, barnyard, little, foxtail, pearl millet | India | Dosa | For GI (n=10), normal healthy participant, For Fasting blood glucose, diabetic participants (n=25), age (35 to 55 yrs) | Randomized cross over trial by feeding each millet dosa for 3 days with interval of 2 days in between. Rice dosa was control. | GI, GL, fasting and post prandial blood glucose |
| Pathak et al., 2000 | Foxtail, barnyard millet | India | Laddu, upma, ladoo | Diabetic participants (n=5), healthy participants (n=5), Age (22 to 27 yrs) | Randomized cross control trial | GI |
| Patil et al., 2015 | Little millet | India | Avalakhi | Healthy participants (n=10), age 30 to 35 yrs) | Self-controlled study | GI, fasting and post prandial blood glucose |
| Poquette et al., 2014 | Sorghum | USA | Muffin | Healthy (n=10), a ge (25.1 ± 4 yrs) | Randomised cross over study. Sorghum muffin was test food and wheat muffin was control food. | GI, fasting and post prandial blood glucose |
| Phanindra et al., 2018 | Foxtail millet | India | Snacks | Diabetic (n=12), age (40 to 60 yrs) | 30 days efficacy study | fasting blood glucose |
| Prasad et al., 2014 | Sorghum | India | Multigrain roti, coarse semolina upma, fine semolina upma, Flakes poha, pasta, biscuits | Healthy (n=10), age (25.6 ± 4.32 yrs) | single cross over design. Sorghum products as intervention food, wheat products as control food fed on different days | GI |
| Rao et al., 2019 | Mixed millet | India | malt | Diabetic individuals (69+73 = 142), intervention 50.06 mean age in yrs, control 51.3 mean age in yrs, | Cross sectional study with long term feeding intervention and observation | Fasting and post prandial blood glucose |
| Ren et al., 2015 | Foxtail millet | China | millet steamed bread, millet pan cake, cooked with water (like rice), porridge | Healthy (n=10), mean age (26 yrs) | Cross over study | GI, insulin index, fasting and post prandial blood glucose level |
| Ren et al., 2018 | Foxtail millet | China | bread | (n=64), age (56.0 ± 7.1 yrs), 27 male and 37 female (67.6% had gestational diabetes) | Self-controlled study conducted for 12 weeks. | Fasting, post prandial blood glucose, post prandial insulin |
| Ruhembe et al., 2014 | White sorghum, finger millet | Tanzania | meal | Healthy (n=10), age (23.75 ± 1.05) | Cross over study | GI, fasting and post prandial blood glucose level |
| Saragih, 2018 | Job’s Tears (Jelai) | Indonesia | Jelai rice | Healthy (n=9), age not indicated | Self-control cross over study | GI, fasting and post prandial blood glucose |
| Shobana et al., 2007 | Finger millet | India | Porridge | Healthy (n=8), age (25 to 52 yrs) | Cross over study | GI |
| Shobana et al., 2018 | Finger millet | India | Upma, vermicelli, snack | Healthy (n=16), age 27.9 ± 3.7 yrs | Cross over study | GI |
| Shubhashini and Ushadevi, 2014. | Pearl millet | India | Roti | Healthy (n=10), age group (20 to 32 yrs) | Controlled clinical trial | GI, fasting and post prandial blood glucose |
| Singh et al., 2020 | Finger millet | India | Meal | Diabetic (n=60), age 44.4 ± 9.160 | self-controlled trial for 12 week study | fasting blood glucose, HbA1c level |
| Sobhana et al., 2020 | Mixed millet | India | Roti | Diabetic participants (n= 47+47), age (40 to 60 yrs) | Randomized controlled clinical trial conducted for 3 months | Fasting blood glucose, HbA1c, fasting insulin level. |
| Surekha et al., 2013 | Barnyard millet | India | Health mix | Healthy participants (n=11), age (25-45 yrs) | Randomized controlled trials | Fasting blood glucose |
| Thakkar and Kapoor, 2007 | Finger millet | India | Roti, idly, upma | Diabetic (n=42), age (40 to 45 yrs) | Case controlled trial | GI, fasting and post prandial blood glucose |
| Thathola et al., 2011 | Foxtail millet | India | biscuits, burfi | Diabetic (n=10), Age (36 to 78 yrs) | Case control clinical trial (30 days) by feeding foxtail millet biscuit to intervention group. Control group didn’t receive any millet and followed regular diet. | GI, serum glucose, HbA1c |
| Thilakavathi and Muthuselvi, 2010 | Kodo, pearl millet | India | Chapattis | Diabetic (n=6+6), age (40 to 60 yrs) | Case control study. whole wheat is control and white bread is reference food. | Fasting and post prandial blood glucose |
| Tiwari and Srivastava, 2017 | Finger millet | India |  | Diabetic (n=15+15), age 40 to 50 yrs | Long term feeding intervention (60 days) | Fasting and post prandial blood glucose |
| Torangatti and Naik, 1999 | Sorghum, pearl millet, foxtail millet, finger millet | India | Millet based meals | Healthy (n=18), diabetic (n=18), age (n=8) 50.3 ± 6.6; female (n=10) 56.1 ± 10.7 | Case control study | GI, fasting and post prandial blood glucose level |
| Ugare et al., 2014 | Barnyard millet | India | upma/rice | Healthy (n=6), diabetic participants (n=9), age (37 to 40 yrs) | Long term feeding study (28 days) | GI, fasting blood glucose |
| Unnikrishnan et al., 2018 | Mixed millet | India | millet bread chicken sandwich | Normal (n=10), | Cross control trial | GI |
| Urooj et al., 2006 | finger millet, sorghum | India | ragi roti, dumpling | Diabetic (n=3), healthy (n=3), age 26 to 60 yrs | Cross over study | GI, fasting and post prandial blood glucose |
| Vedamanickam et al., 2020 | Mixed millet | India | Meal | 80+70 = 150: Intervention (n=80), Control group (n=70), diabetic individuals | Cross sectional study conducted with long term observation | Fasting and post prandial blood glucose |
| Wahlang et al., 2018 | Foxtail millet | India | rice | Healthy (n=6), Age (22 to 28 yrs) | Cross over study. | GI, fasting and post prandial blood glucose |
