# Supplementary figures and images for: A Systematic Review and Meta-Analysis of the Potential of Millets for Managing and Reducing the Risk of Developing Diabetes Mellitus
Source: Front Nutr. 2021 Jul 28;8:687428. doi: 10.3389/fnut.2021.687428 (PMC8355360; doi:10.3389/fnut.2021.687428)

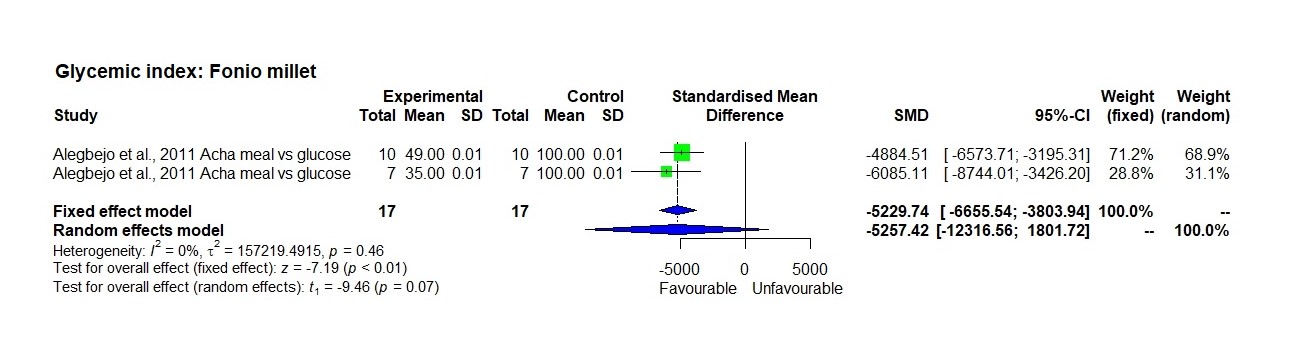

Supplement: Supplementary file 2 [file Image_1.jpeg]

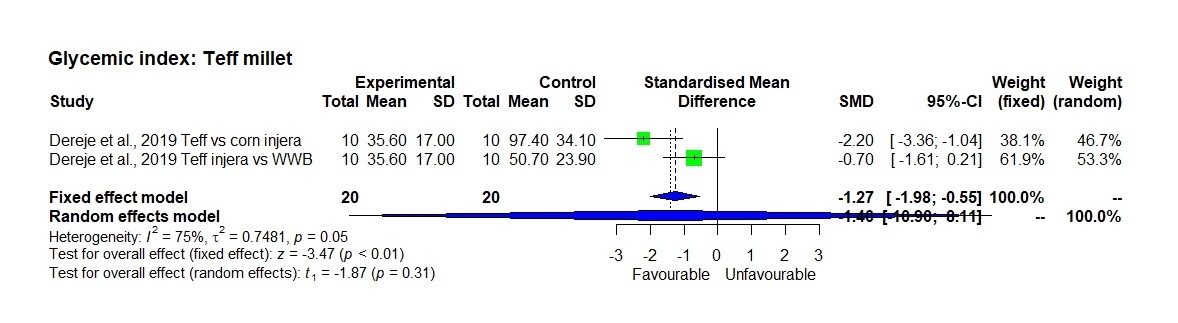

Supplement: Supplementary file 3 [file Image_2.jpeg]

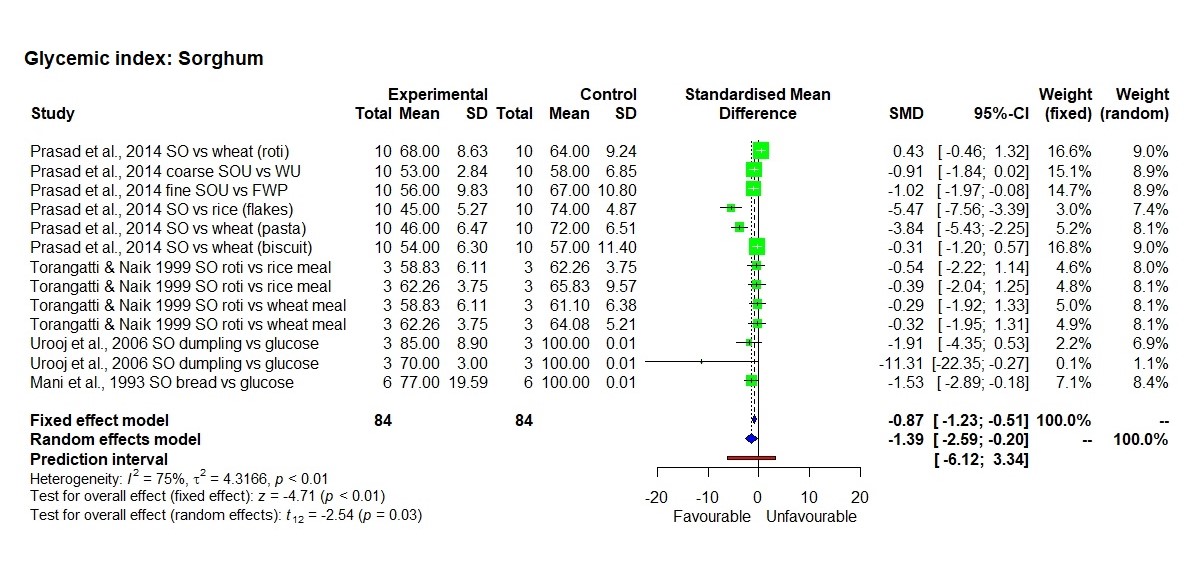

Supplement: Supplementary file 4 [file Image_3.jpeg]

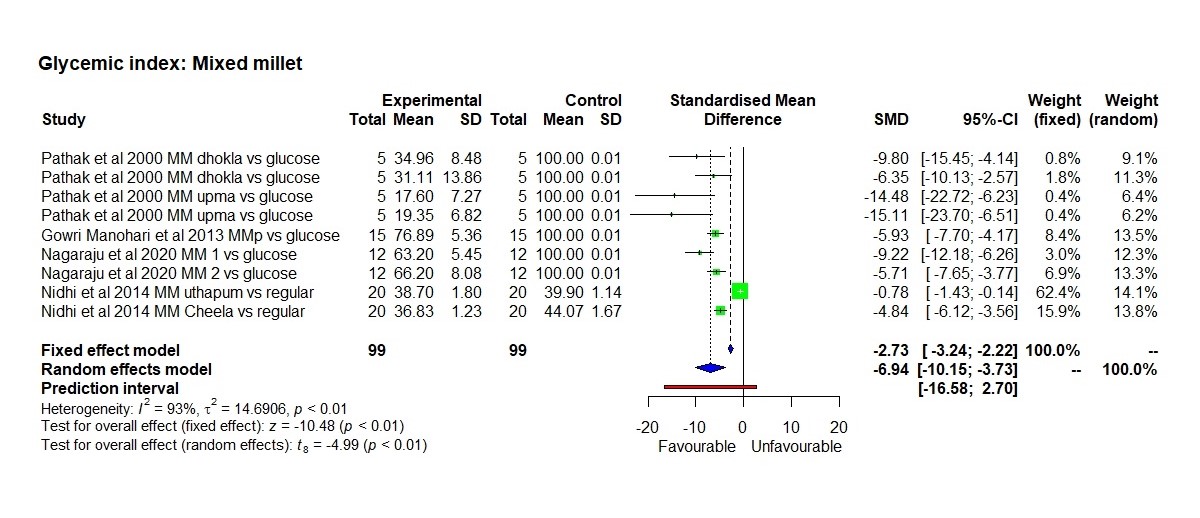

Supplement: Supplementary file 5 [file Image_4.jpeg]

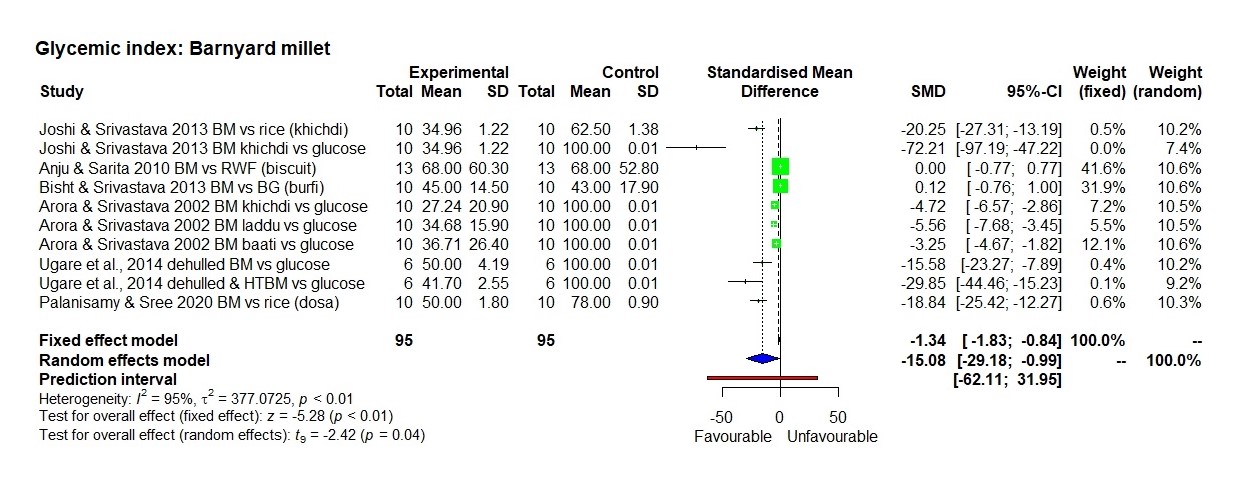

Supplement: Supplementary file 6 [file Image_5.jpeg]

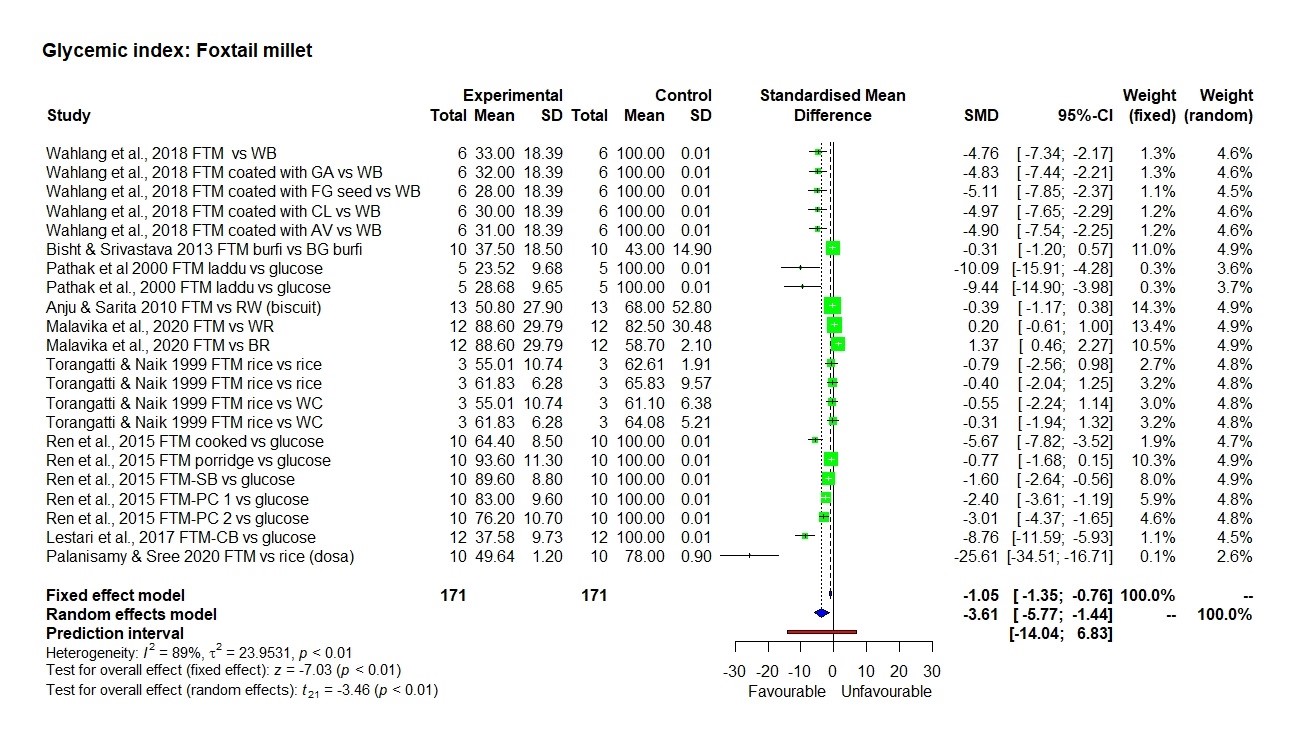

Supplement: Supplementary file 7 [file Image_6.jpeg]

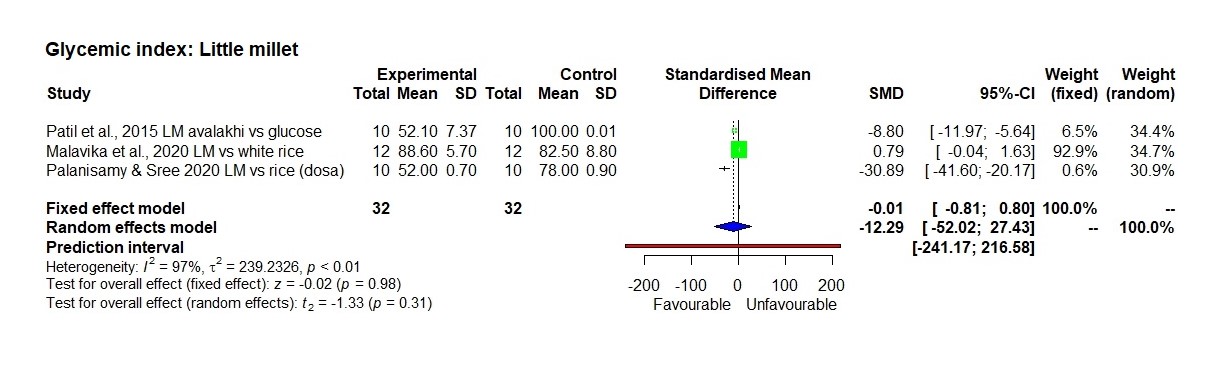

Supplement: Supplementary file 8 [file Image_7.jpeg]

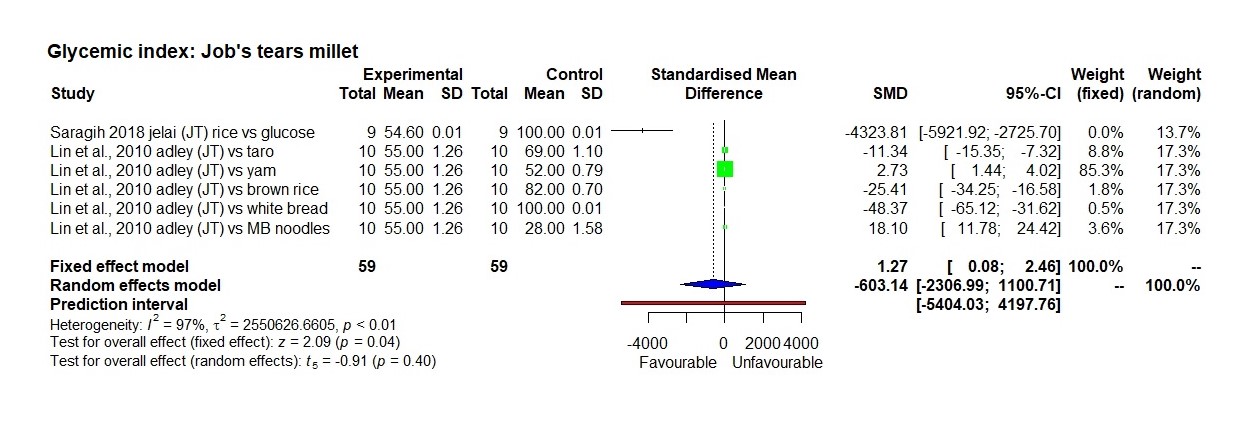

Supplement: Supplementary file 9 [file Image_8.jpeg]
